# *C9orf72* Repeat Expansion Induces Metabolic Dysfunction in Human iPSC‐Derived Microglia and Modulates Glial‐Neuronal Crosstalk

**DOI:** 10.1002/glia.70080

**Published:** 2025-09-01

**Authors:** Marika Mearelli, Insa Hirschberg, Christin Weissleder, Carmela Giachino, María José Pérez, Malvina Dubroux, Francesca Provenzano, Mafalda Rizzuti, Linda Ottoboni, Udit Sheth, Tania F. Gendron, Stefania Corti, Michela Deleidi

**Affiliations:** ^1^ Hertie Institute for Clinical Brain Research University of Tübingen Tübingen Germany; ^2^ German Center for Neurodegenerative Diseases (DZNE) Tübingen Germany; ^3^ Mechanisms and Therapy of Genetic Brain Diseases Institut Imagine INSERM UMR1163 Paris France; ^4^ Neurology Unit, Foundation IRCCS Cà Granda Ospedale Maggiore Policlinico Milan Italy; ^5^ Dino Ferrari Centre, Department of Pathophysiology and Transplantation (DEPT) Università Degli Studi di Milano Milano Italy; ^6^ Department of Neuroscience Mayo Clinic Jacksonville Florida USA; ^7^ Mayo Clinic Graduate School of Biomedical Sciences Mayo Clinic Jacksonville Florida USA; ^8^ Neuromuscular and Rare Diseases Unit, Department of Neuroscience Fondazione IRCCS Cà Granda Ospedale Maggiore Policlinico Milan Italy

**Keywords:** amyotrophic lateral sclerosis/frontotemporal dementia, *C9orf72*, glial‐neuronal communication, immune system, induced pluripotent stem cells, microglia

## Abstract

The *C9orf72* hexanucleotide repeat expansion mutation is the most common genetic cause of amyotrophic lateral sclerosis (ALS) and frontotemporal dementia, but its cell type‐specific effects on energy metabolism and immune pathways remain poorly understood. Using induced pluripotent stem cell (iPSC)‐derived motor neurons, astrocytes, and microglia from *C9orf72* patients and their isogenic controls, we investigated metabolic changes at the single‐cell level under basal and inflammatory conditions. Our results showed that microglia are particularly susceptible to metabolic disturbances. While *C9orf72* motor neurons exhibited impaired mitochondrial respiration and reduced ATP production, *C9orf72* microglia presented pronounced increases in glycolytic activity and oxidative stress, accompanied by the upregulation of the expression of key metabolic enzymes. These metabolic changes in microglia were exacerbated by inflammatory stimuli. To investigate how these changes affect the broader cellular environment, we developed a human iPSC‐derived triculture system comprising motor neurons, astrocytes, and microglia. This model revealed increased metabolic activity in all cell types and highlighted that microglia‐driven metabolic reprogramming in astrocytes contributes to the vulnerability of motor neurons under inflammatory conditions. Our findings highlight the central role of microglia in driving metabolic dysregulation and intercellular crosstalk in ALS pathogenesis and suggest that targeting metabolic pathways in immune cells may provide new therapeutic avenues.

## Graphical abstract

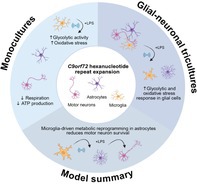

*C9orf72* microglia display heightened glycolysis and oxidative stress, driving astrocytic reprogramming that impacts motor neurons. iPSC tricultures uncover key glial–neuronal interactions in *C9orf72* pathology.

**Abbreviations**
ALS: amyotrophic lateral sclerosis
CNS: central nervous system
ECAR: extracellular acidification rate
FTD: frontotemporal dementia
GLUT1: glucose transporter 1
G6PD: glucose 6‐phosphate dehydrogenase
HK1: hexokinase‐1
iPSCs: induced pluripotent stem cells
Met‐Flow: metabolic flow cytometry
MNs: motor neurons
OCR: oxygen consumption rate
PRDX2: peroxiredoxin 2

## 1 Introduction

Amyotrophic lateral sclerosis (ALS) is the most common adult‐onset motor neuron disease and is characterized by the progressive degeneration and death of motor neurons (MNs) in the cerebral cortex, brainstem, and spinal cord. ALS pathogenesis is multifactorial and involves diverse mechanisms that contribute to the selective vulnerability and loss of MNs. Traditionally considered a disease driven primarily by the intrinsic susceptibility of MNs to autonomous cell death, increasing evidence highlights the importance of non‐cell‐autonomous mechanisms in MN degeneration (Ilieva et al. 2009). In particular, interactions among neurons, astrocytes, and immune cells have emerged as pivotal contributors to disease onset and progression (Peng et al. 2020). ALS is characterized by widespread neuroinflammation, including astrogliosis, microglial activation, and peripheral immune cell infiltration at sites of neuronal degeneration. Notably, autoimmune diseases often precede the onset of ALS/frontotemporal dementia (FTD) (Turner et al. 2013; Miller et al. 2016), and microglial activation at disease onset is correlated with the rate of motor decline (Turner et al. 2004; Zürcher et al. 2015).

Although most ALS cases are sporadic, several gene mutations are associated with familial forms of the disease. Among these, the expansion of a non‐coding hexanucleotide repeat (GGGGCC) in the *C9orf72* gene is the most common genetic cause of ALS and FTD (Renton et al. 2011; DeJesus‐Hernandez et al. 2011). This mutation accounts for 20%–40% of familial ALS cases and 2%–8% of sporadic cases, with a higher prevalence among individuals of European descent (Majounie et al. 2012). The high frequency of *C9orf72* mutations in both ALS and FTD has stimulated extensive research into its role in neuronal damage and degeneration. A critical question is whether and how *C9orf72* contributes to the risk and progression of the disease. *C9orf72* is ubiquitously expressed, including in peripheral myeloid cells and microglia (Rizzu et al. 2016). The loss of *C9orf72* in mice enhances proinflammatory responses in peripheral myeloid cells and microglia (O'Rourke et al. 2016; Sudria‐Lopez et al. 2016). Emerging evidence also highlights the critical role of *C9orf72* in intercellular communication and metabolic regulation, with *C9orf72* being a key regulator of mitochondrial function and cellular energy homeostasis (Wang et al. 2021). Interestingly, compared with control astrocytes, ALS‐induced astrocytes display a dysregulated metabolic profile characterized by a significant loss of metabolic flexibility (Allen et al. 2019). Additionally, *C9orf72* deficiency has been associated with impairments in autophagy and lysosomal function, processes essential for cellular homeostasis and the clearance of pathogenic aggregates capable of propagating between cells (Amick et al. 2016). The *C9orf72*‐mediated regulation of inflammatory signaling in macrophages and microglia may influence the local metabolic microenvironment, potentially disrupting the neuron–glia interactions critical for disease progression. Such disruptions may exacerbate energy deficits in MNs, further increasing their susceptibility to degeneration. Understanding how *C9orf72* affects cell type‐specific metabolism and intercellular communication is essential for identifying novel therapeutic strategies for ALS and related diseases.

To address these issues, we generated *C9orf72* mutant human induced pluripotent stem cell (iPSC)‐derived MNs and glial cells (microglia and astrocytes) in both monoculture and co‐culture systems. We used spectral flow cytometry for high‐dimensional single‐cell analysis, integrating metabolic flow cytometry (Met‐Flow) to comprehensively assess cell type‐specific cellular metabolism. This study provides an in‐depth metabolic analysis of human *C9orf72* mutant iPSC‐derived MNs and glia, providing novel insights into *C9orf72*‐related metabolic dysregulation, neuron–glia interactions, and their contributions to disease pathogenesis.

## 2 Methods

### 2.1 Induced Pluripotent Stem Cell (iPSC) Culture

The following human *C9orf72* iPSC lines and corresponding isogenic controls were used in this study: CS52iALS‐C9nxx/CS52iALS‐C9n6.ISOxx and CS29iALS‐C9nxx/CS29iALS‐C9n1.ISOxx (obtained from Cedars‐Sinai, Los Angeles, CA, USA) and BS6/BS62H9 (Selvaraj et al. 2018). Human iPSCs were maintained on Matrigel (Corning) in mTeSR Plus medium (STEMCELL Technologies) supplemented with penicillin–streptomycin (P/S, Merck Millipore). Cultures were routinely tested for mycoplasma contamination using a Venor GeM Classic detection kit (Minerva Biolabs GmbH).

### 2.2 Differentiation of Human iPSCs Into Motor Neurons

MN differentiation was performed in accordance with a previously established protocol (Maury et al. 2015). Briefly, confluent iPSC cultures were detached from Matrigel‐coated plates and dissociated into single cells using Accutase (Stem Cell Technologies) at 37°C for 5 min. The resulting single cells were seeded at 31,000 cells/cm^2^ on Matrigel‐coated 6‐well plates for differentiation in a 1:1 mixture of Neurobasal medium (Gibco) and Advanced DMEM/F12 (Gibco) supplemented with 1% P/S, 1% GlutaMAX (Gibco), 100 μM β‐mercaptoethanol (Sigma‐Aldrich), 1% B27 (Gibco), 1% N2 (Gibco), 10 μM ascorbic acid (Sigma Aldrich), 40 μM SB‐431542 (Selleck Chemicals), 0.2 μM LDN‐193189 (Axon MedChem) and 5 μM Y‐27632 (Selleck Chemicals). Medium was supplemented with 3 μM CHIR99021 (Sigma Aldrich), 0.1 μM retinoic acid (MedChem Express), and 500 nM Smoothened Agonist (Merck Millipore) the day after. On day 8, 10 ng/mL BDNF (Peprotech) and 10 ng/mL GDNF (Peprotech) were added to replace CHIR99021, LDN‐193189, and SB‐431542. On day 9, the medium was supplemented with 20 μM DAPT (Selleck Chemicals) in addition to the aforementioned factors. On day 11, MN progenitors were dissociated using Accutase at 37°C for 5 min and plated at 31,000 cells/cm^2^ on Matrigel‐coated 6‐well plates. On day 17, the medium was replaced with the maturation medium with 2.5 μM ascorbic acid and 10 ng/mL CNTF (Immunotools).

### 2.3 Differentiation of Human iPSCs Into Microglia

iPSCs were differentiated into microglia following an established protocol with minor modifications (Haenseler et al. 2017). EBs were formed using AggreWell 800 plates (STEMCELL Technologies) and cultured in mTeSR Plus supplemented with 50 ng/mL BMP4 (ImmunoTools), 50 ng/mL VEGF (ImmunoTools) and 20 ng/mL SCF (ImmunoTools) for 4 days, with 75% of the medium changed daily. On day 4, the EBs were harvested and transferred to a 6‐well plate (12–16 EBs/well) in X‐VIVO 15 (Lonza) supplemented with 25 ng/mL IL‐3 (ImmunoTools), 100 ng/mL M‐CSF (ImmunoTools), 2 mM GlutaMAX, 1% P/S, and 0.055 mM β‐mercaptoethanol, with weekly medium changes. After 3–4 weeks, floating cells were collected and seeded at 100,000 cells/cm^2^ on Matrigel‐coated plates in Advanced DMEM/F‐12 supplemented with 1% N2, 1% GlutaMAX, 1% P/S, 0.055 mM β‐mercaptoethanol, 100 ng/mL M‐CSF, 100 ng/mL IL‐34 (PeproTech) and 10 ng/mL GM‐CSF (ImmunoTools), with the medium changed twice weekly.

### 2.4 Differentiation of Human iPSCs Into Astrocytes

Human iPSCs were differentiated into astrocytes in accordance with the protocol described by Tcw et al. (Tcw et al. 2017). To detach iPSC colonies, the cells were treated with 1 mg/mL collagenase (Thermo Fisher Scientific) for 1 h. After collagenase treatment, the cells were suspended in EB medium and transferred to untreated polystyrene plates for 7 days, after which the medium was changed daily. The EB medium consisted of DMEM/F12 (Gibco), 20% knockout serum replacement (Gibco), 1% GlutaMAX, 1% MEM non‐essential amino acids (NEAAs, Gibco), 100 μM β‐mercaptoethanol, 2 μM dorsomorphin (Tocris) and 2 μM A‐83 (Tocris). After 7 days, the EB medium was replaced with neural induction medium consisting of DMEM/F12, 1% N2, 1% B27, 1% NEAAs, 1% GlutaMAX, 2 μg/mL heparin (Sigma Aldrich) and 2 μM cyclopamine (Tocris). On day 7, floating EBs were transferred to Matrigel‐coated 6‐well plates to form neural tube‐like rosettes, which were maintained for 15 days, with the medium changed every other day. On day 22, the rosettes were mechanically picked and transferred to low‐attachment plates (Corning) in neural induction medium supplemented with B27. Neural precursor spheres were differentiated into astrocytes as described by Tcw et al. with minor modifications. Spheres were dissociated with Accutase for 10 min at 37°C and seeded at 15,000 cells/cm^2^ on Matrigel‐coated plates in astrocyte medium (ScienCell, astrocyte medium containing 2% fetal bovine serum (FBS), astrocyte growth supplement and 10 U/mL P/S). Starting on day 2, the cells were fed every 48 h for 20–30 days. Astrocytes were split at 90%–95% confluence (approximately every 6–7 days) and re‐seeded as single cells in astrocyte medium at the same initial seeding density (15,000 cells/cm^2^).

### 2.5 Hexanucleotide Repeat Expansion Analysis

The presence of the hexanucleotide repeat expansion was assessed through a specific Repeat‐Primed PCR assay design to profile repeat sequences in the *C9orf72* gene using the AmplideX PCR/CE *C9orf72* Kit (Bio‐techne, Asuragen). PCR products (2 μL each) were diluted 1:5 in water, mixed with 10 μL of formaldehyde, and analyzed on a 3100 DNA Analyzer (Applied Biosystems) in the presence of the GeneScan 500 LIZ size standard (Applied Biosystems). Fragment analysis was carried out using GeneMapper software (Applied Biosystems).

### 2.6 Real Time PCR

Total RNA was extracted using the ReliaPrep RNA Cell Kit (Promega) and reverse‐transcribed using the SuperScript VILO Master Mix (Thermo Fisher Scientific). Real Time PCR experiments were set up using the SYBR Green PCR Master Mix (Thermo Fisher Scientific) and the specific primers for *C9orf72* transcript variants (3 different isoforms), Stathmin‐2 (*STMN2*, both full‐length and truncated isoforms) and *UNC13A* (Table S1). Plates were run on the 7500 Real Time PCR System (Applied Biosystem). The expression levels of selected candidate genes were normalized to the average levels of *GAPDH* and referred to control samples by means of the ΔΔCt method. Data are presented as the means of triplicates. Only results with Ct < 35 were taken into consideration for analysis. Gene expression analysis was performed on GraphPad Prism 10.

### 2.7 Dipeptide Repeat Analysis

Samples were first weighed, and lysis was performed by adding Co‐IP buffer at a ratio of 1:10 (buffer: tissue, w/v). The buffer consisted of 50 mM Tris–HCl (pH 7.4), 300 mM NaCl, 1% Triton X‐100, 5 mM EDTA, 1 mM phenylmethylsulfonyl fluoride, Phosphatase Inhibitor Cocktail (Cat. #B1500, Bimake), and Protease Inhibitor Cocktail (Cat. #539131, EMD Millipore). Cell pellets were homogenized by pipetting, thoroughly mixed, and SDS was added to a final concentration of 2%, followed by vigorous vortexing and subsequent sonication. Lysates were centrifuged at maximum speed for 20 min at room temperature, and the resulting supernatants were transferred to fresh tubes. Protein concentration was determined using the BCA assay. Meso Scale Discovery technology was employed to quantify poly‐GA and poly‐GP detection as previously described (Gendron et al. 2017; Andrade et al. 2020). In brief, an affinity‐purified monoclonal poly‐GA antibody (clone 5E9, Cat. #MABN889, Millipore Sigma) was used as both the capture antibody (5E9, 2 μg/mL) and the detection antibody (sulfo‐tagged 5E9, 2 μg/mL) for the poly(GA) immunoassay; an affinity‐purified monoclonal poly‐GP antibody (TALS 828.179) generated by Target ALS was used as both the capture antibody (biotinylated TALS 828.179, 2 μg/mL) and the detection antibody (sulfo‐tagged TALS 828.179, 2 μg/mL) for the poly(GP) immunoassay. Samples were diluted in Tris‐buffered saline to 20–35 μg protein per well and tested in duplicate. Positive and negative controls were tested in conjunction with the samples. Response values corresponding to the intensity of emitted light upon electrochemical stimulation of the assay plate were acquired using the Meso Scale Discovery QUICKPLEX SQ120. Poly‐GA and poly‐GP levels are presented in arbitrary units.

### 2.8 Western Blotting

Proteins were extracted using 0.5% NP40‐PBS protein extraction buffer containing protease and phosphatase inhibitors (Roche) on ice. The suspension was then centrifuged at 13,000 rpm at 4°C for 15 min. The protein concentration of the supernatant was determined using a Pierce BCA Protein Assay Kit (Thermo Fisher Scientific). A total of 20–50 μg of the protein lysate was loaded onto a polyacrylamide gel (SurePAGE, Bis‐Tris, 10 × 8, 4%–12%, GenScript) and transferred to a 0.2–0.45 μm PVDF membrane (Millipore). Blots were blocked with 5% milk powder in TBS + 0.1% Tween‐20 (TBST) and incubated with primary antibodies in milk blocking solution overnight at 4°C (C9orf27: 1:500, Cat. #GTX632041, Genetex; ACTB, 1:10000, Cat. #A5441, Sigma Aldrich; GAPDH: 1: 10000, Cat. #sc‐47724, Santa Cruz). This step was followed by incubation with corresponding HRP‐conjugated secondary antibodies (Sigma Aldrich) for 1 h at room temperature. Visualization of proteins was carried out using ECL Western Blotting Detection Reagent (Pierce, Thermo Fisher Scientific) and visualized in ChemiDoc MP imaging system (Bio‐Rad).

### 2.9 Single‐Cell RNA Sequencing Library Generation

To obtain single‐cell suspensions, iPSC‐derived microglia were enzymatically dissociated using Accutase (15 min, 37°C), followed by filtration through a 40 μm cell strainer. Cells were resuspended in PBS supplemented with 0.04% bovine serum albumin at a target concentration of 1000 cells/μL. Only preparations with cell viability exceeding 95% were processed. Single‐cell libraries were prepared using the Chromium Next GEM Single Cell 3′ Reagent Kit v3.1 (Cat. #1000128, 10× Genomics) in accordance with the manufacturer's recommendations. Libraries were pooled and sequenced in paired‐end mode on an Illumina NovaSeq 6000 system (SP Flow Cell) with a minimum of 300 million reads per library. Sequencing was conducted by GENEWIZ GmbH (Leipzig, Germany).

### 2.10 Preprocessing and Analysis of Single‐Cell Transcriptomic Data

Raw base call files were processed using the Cell Ranger pipeline from 10× Genomics to demultiplex samples, align reads, and generate filtered gene‐barcode matrices. The downstream analysis was performed using the Seurat package (v4.1.0, RRID: SCR_007322) in R. A total of 4249 cells for the mutant and 6656 cells for the isogenic control microglia passed initial quality control. Cells with fewer than 2000 UMIs, fewer than 500 detected genes, or a mitochondrial gene content greater than 20% were excluded. Genes not detected in at least ten cells were also removed from the dataset. Doublets were predicted and filtered using the DoubletFinder package (v3, RRID: SCR_018771). SCTransform (v0.4.1, RRID: SCR_022146) was applied for normalization, including regression of cell cycle and mitochondrial content. Data integration across samples was performed using the Harmony algorithm (v1.2.0, RRID: SCR_022206). Dimensionality reduction was carried out using principal component analysis, and the first 20 principal components were used to build a shared nearest‐neighbor graph. Clustering was performed at a resolution of 0.6, and the results were visualized with Uniform Manifold Approximation and Projection. This approach resolved 14 distinct cell clusters. Cell expression in a specific cluster was analyzed using Seurat's FindMarkers function (parameters: min.pct = 0.25, logfc.threshold = 0.25). Functional enrichment of differentially expressed genes was performed using standard annotation tools, referencing the gene annotation file available at: https://raw.githubusercontent.com/hbctraining/scRNA‐seq/master/data/annotation.csv. The top 100 markers were used to identify the most significant genes associated with clusters unique to the mutant condition (log2FC > 0.5, *p*‐value < 0.05).

### 2.11 Determination of ATP Levels

The levels of intracellular ATP was determined using an ATP determination kit (Cat. #A22066, Thermo Fisher Scientific) in accordance with the manufacturer's instructions. The amount of ATP in each sample was calculated from standard curves and normalized to the total protein concentration.

### 2.12 Seahorse XFp Metabolic Flux Analysis

The OCR and ECAR were measured using an XFp Extracellular Flux Analyzer (Agilent Technologies). iPSC‐derived MNs, astrocytes, and microglia were cultured on XFp Cell Culture Miniplates (Agilent Technologies) at a density of 100,000 MNs/well for 10 days, 25,000 astrocytes/well for 5 days, or 80,000 microglia/well for 7 days. The OCR and ECAR were measured in freshly prepared medium consisting of phenol‐free DMEM (pH 7.4) supplemented with 1 mM GlutaMAX. Oxygen consumption was analyzed after the sequential injection of oligomycin, carbonyl cyanide m‐chlorophenylhydrazone (CCCP), and antimycin A/rotenone. Glycolytic function was evaluated after the sequential injection of glucose, oligomycin, and 50 mM 2‐deoxy‐D‐glucose (all from Sigma Aldrich); three measurements were obtained for each condition, each lasting 3 min. The values were normalized to the protein concentration as measured by a Pierce BCA Protein Assay Kit (Thermo Fisher Scientific).

### 2.13 Measurement of Reactive Oxygen Species

Intracellular reactive oxygen species levels were measured using the 2′,7′‐dichlorodihydrofluorescein diacetate probe (Cat. #ab113851, Abcam) following the manufacturer's instructions. Cells were treated with 2′,7′‐dichlorodihydrofluorescein diacetate (25 μM) for 45 min at 37°C. After incubation, cells were harvested and the fluorescence intensity was quantified using a Bio‐Rad plate reader (Ex/Em = 485/535 nm).

### 2.14 Human iPSC Tricultures

For the triculture of iPSC‐derived astrocytes, MNs and microglia, the differentiation protocols were followed as described above with minor modifications. Astrocytes were differentiated as described and seeded at 10,000 cells/cm^2^ into Matrigel‐coated 6‐well plates on day 0 and maintained in astrocyte medium with 0.5% FBS for 3 days. On day 3, MN precursors were seeded at 52,000 cells/cm^2^ on pre‐plated astrocytes (day 11 of the MN differentiation protocol). The medium was then changed on day 11 to MN medium supplemented with 4% astrocyte medium without FBS. On day 9 (day 17 of MN differentiation protocol), microglial precursor cells were harvested and seeded onto co‐cultured astrocytes and MNs at 31,000 cells/cm^2^. The medium was replaced in accordance with the MN maturation protocol and supplemented with 4% astrocyte medium without FBS, 100 ng/mL M‐CSF, 100 ng/mL IL‐34, and 10 ng/mL GM‐CSF. Approximately 50%–66% of the medium was replaced on days 11 and 13. On day 15, the cells were ready for subsequent experiments.

### 2.15 Immunocytochemistry and Image Analysis

Cells were fixed in 4% paraformaldehyde in PBS (w/v) for 15 min, washed with PBS, and blocked with PBS containing 0.1% Triton X‐100 (PBST) supplemented with 10% normal goat serum (NGS) or 10% normal donkey serum (NDS) for 1 h. The cells were then incubated overnight at 4°C with the primary antibody of interest, which was diluted in PBST containing 5% NGS or NDS. After three 10 min washes in 1× PBS, the appropriate species‐specific Alexa Fluor 488/568/647‐conjugated secondary antibody (Invitrogen) was applied to the cells for 2 h at room temperature in PBST containing 5% serum. Next, the cells were washed once for 10 min in 1× PBS, followed by 10 min of DAPI staining (1:10,000) and three additional 10 min washes. The cells were mounted with fluorescent mounting medium (Agilent Technologies). The primary antibodies used were rabbit anti‐HB9 polyclonal antibody (1:500, Cat. #HPA‐071717, Sigma Aldrich), rabbit anti‐CHAT (1:200, Cat. #AB144P, Merck Millipore), mouse anti‐tubulin β III (TUBB3, 1:1000, Cat. #801202, BioLegend), rabbit anti‐TUBB3 (1:1000, Cat. #903401, BioLegend), mouse anti‐PU1 (1:500, Cat. #658002, BioLegend), rabbit anti‐IBA1 (1:500, Cat. #016‐2001, WAKO), chicken anti‐GFAP (1:2000, Cat. #829401, BioLegend), rabbit anti‐GFAP (1:500, Cat. #Z0334, Dako), rabbit anti‐KI67 (1:250, Cat. #MA5‐14520, Invitrogen), chicken anti‐MAP2 (1:10000, Cat. #822501, BioLegend), mouse anti‐GLAST (1:500, Cat. #130–132‐738, Miltenyi Biotech), mouse anti‐S100B (1:1000, Cat. #S2532, Sigma‐Aldrich) and Phalloidin‐iFluor 594 (1:5000, Cat. #ab176757, Abcam). For LPS treatment, 100 ng/mL LPS (ultrapure LPS from *E. coli* 0111:B4, InvivoGen) was added to mono‐ or tricultures 24 h prior to sample collection. Where indicated, conditioned medium was collected from control and *C9orf72* tricultures and tricultures stimulated for 24 h with 100 ng/mL LPS. The conditioned medium was filtered and then used for MN survival assays. Neuronal counts were quantified by counting the number of TUBB3 or MAP2‐positive neurons in 10 randomly selected fields per sample. MN survival was calculated for each condition relative to the average value of the isogenic control under basal condition. For the analysis of cell proliferation, KI67‐positive cells were counted in PU1‐positive microglia and normalized to the integrated density of DAPI immunostaining. KI67‐positive cells without detectable PU1 expression were considered proliferating astrocytes. Each condition was tested in a minimum of three independent experiments. Images were analyzed with Fiji‐ImageJ version 2.3.0/1.53q.

### 2.16 Flow Cytometry

Cells were detached with Accutase, resuspended in 100 μL of PBS, stained with Zombie NIR Fixable Viability Dye (BioLegend), incubated with Fc block (BioLegend) for 15 min, counted, and incubated for 30 min in the dark with fluorophore‐conjugated antibodies. After washing, the cells were fixed in 4% PFA (15 min, room temperature, dark) after permeabilization with 0.1% Triton X‐100 and 1% FBS in PBS for 15 min at room temperature in the dark. The cells were then stained with primary antibodies for intracellular staining in permeabilization/blocking buffer and incubated for 30 min on ice, protected from light, followed by a second incubation for 30 min on ice with secondary antibodies. After final washes, the cells were resuspended in PBS containing 2% FBS and transferred to 5 mL round bottom tubes for flow cytometry. Filter cap tubes were used for MN samples to avoid clogging the instrument. The samples were run on a BD LSRFortessa and analyzed using FlowJo software. The following antibodies were used for flow cytometry: anti‐HB9 (1:100, Cat. #BS‐11320R, Thermo Fisher Scientific), anti‐CD11B Brilliant Violet 785 (clone ICRF44, 1:40, Cat. #301346, BioLegend), anti‐CD206 Brilliant Violet 605 (clone 15‐2, 1:50, Cat. #321139, BioLegend); anti‐HLA‐DR Peridinin‐Chlorophyll‐Protein/Cyanine 5.5 (1:50, Cat. #307629, Biolegend), anti‐CD86 Pacific Blue (clone IT2.2, 1:50, Cat. #305423, BioLegend), anti‐CX3CR1 Phycoerythrin (PE)/Cyanine 7 (clone 2A9‐1, 1:100, Cat. #341611, BioLegend), anti‐ARGI1 PE (clone 14D2C43, 1:200, Cat. #369703, BioLegend), anti‐CD49F PE/Cyanine 7 anti‐human/mouse (clone GoH3, 1:50, Cat. #313622, BioLegend), anti‐HLA‐E Peridinin‐Chlorophyll‐Protein/Cyanine 5.5 (clone 3D12, 1:100, Cat. #342609, BioLegend), anti‐GLAST (1:100, Cat. #130‐118‐483, Miltenyi), CoraLite 488‐conjugated anti‐PRDX2 (Clone 3H6C4, 1:50, Cat. #CL488‐60202, ProteinTech), DyLight 650 anti‐GLUT1 (clone GLUT1/2475, 1:50, Cat. #NBP2‐75786C, Novus Biologicals), Immunotag AF405 anti‐G6PD monoclonal antibody (1:50, Cat. #ITM0292, G‐Biosciences), and Immunotag AF594 anti‐HK I monoclonal antibody (1:50, Cat. #ITM0348, G‐Biosciences). For the Met‐Flow experiments, MNs were labeled with 1 μM CellTrace Yellow dye (Cat. #C34573, Thermo Fisher Scientific) prior to being seeded onto precoated astrocytes. Precursors were detached using Accutase, counted, and stained in serum‐free conditions with 1× PBS for 20 min at 37°C with 5% CO₂. The staining process was halted by adding 20% FBS for 5 min. The cells were subsequently centrifuged and resuspended in MN medium supplemented with 4% astrocyte medium. High‐dimensional analysis was performed using Fast Fourier Transform‐accelerated interpolation‐based t‐distributed Stochastic Neighbor Embedding (FitSNE) in FlowJo (BD, version 10.6.1). FitSNE was applied to a down‐sampled dataset of 10,000–20,0000 cells per sample. The histograms adjacent to the FitSNE plots show the number of fluorescence channels on the y‐axis and the biexponential fluorescence intensity of each marker on the x‐axis.

### 2.17 Statistics

Statistical differences among groups were evaluated using GraphPad Prism Version 10.2.1. All experiments were performed at least in triplicate, and the quantitative data are presented as means ± SEMs. Sample sizes were chosen based on previous studies. All samples were included in the analysis. Blinding was not performed in this study. Differences among groups were assessed using an unpaired two‐tailed Student's *t* test or ANOVA for multiple comparisons, as indicated in the figure legends.

## 3 Results

### 3.1 The *C9orf72* Repeat Expansion Mutation Exerts a Cell Type‐Specific Effect on Energy Metabolism in Human iPSC‐Derived Motor Neurons, Astrocytes, and Microglia

**FIGURE 1:**
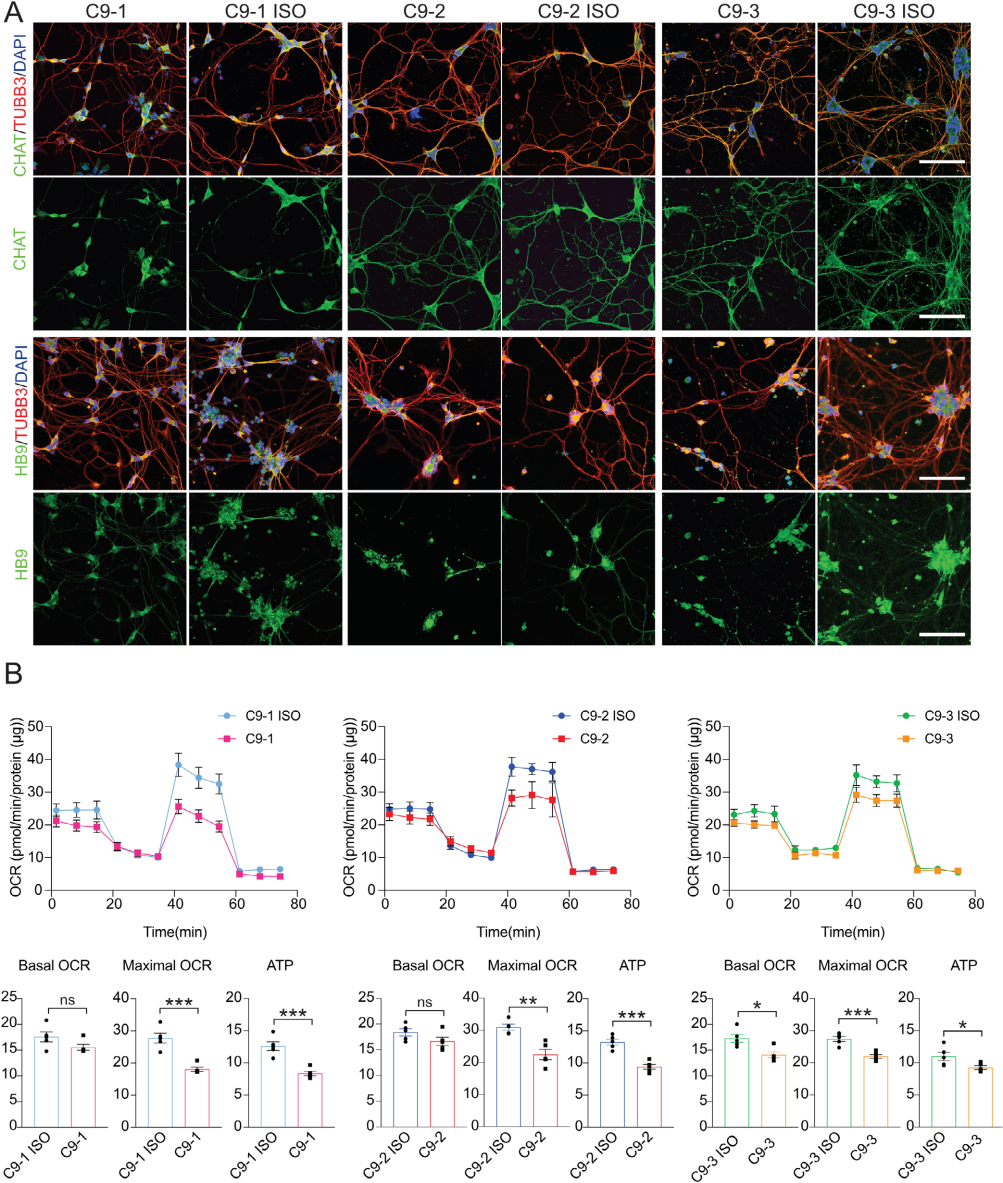
Differentiation and mitochondrial bioenergetic characterization of *C9orf72* and paired isogenic control iPSC‐derived motor neurons. (A) Representative confocal images showing the immunocytochemical staining of CHAT (green) or HB9 (green) with TUBB3 (red) and nuclei (DAPI; blue) in *C9orf72* (C9) and paired isogenic control (C9‐ISO) iPSC‐derived motor neurons (MNs). Scale bars, 100 μm. (B) Mitochondrial respiration in C9 and C9‐ISO MNs. Oxygen consumption rates (OCRs) were measured over time after the sequential addition of oligomycin, CCCP, and rotenone/antimycin A. Values for the basal OCR, ATP‐linked OCR, and maximal OCR are expressed as pmol/min/protein (μg). Mean ± SEM; unpaired two‐tailed t test; *p < 0.05, **p < 0.01, and ***p < 0.001; *n* = 5 independent experiments.

**FIGURE 2:**
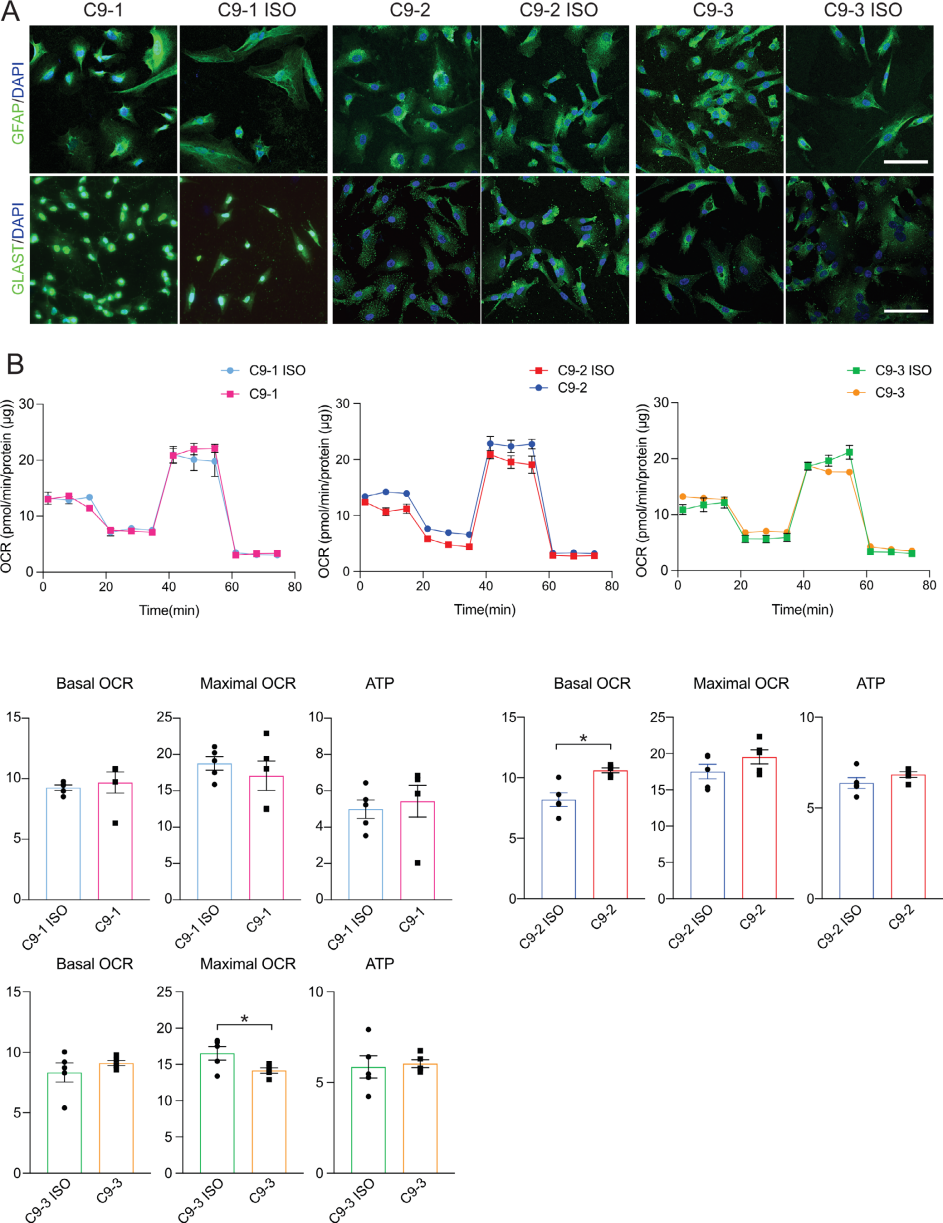
Differentiation and mitochondrial bioenergetic characterization of *C9orf72* and paired isogenic control iPSC‐derived astrocytes. (A) Representative confocal images showing the immunocytochemical staining of GFAP (green) or GLAST (green) with nuclei (DAPI; blue) in *C9orf72* (C9) and paired isogenic control (C9‐ISO) iPSC‐derived astrocytes. Scale bars, 100 μm. (B) Mitochondrial respiration in C9 and C9‐ISO astrocytes. Oxygen consumption rates (OCRs) were measured over time after the sequential addition of oligomycin, CCCP, and rotenone/antimycin A. Values for the basal OCR, ATP‐linked OCR, and maximal OCR are expressed as pmol/min/protein (μg). Mean ± SEM; unpaired two‐tailed t test; *p < 0.05; *n* = 5 independent experiments.

**FIGURE 3:**
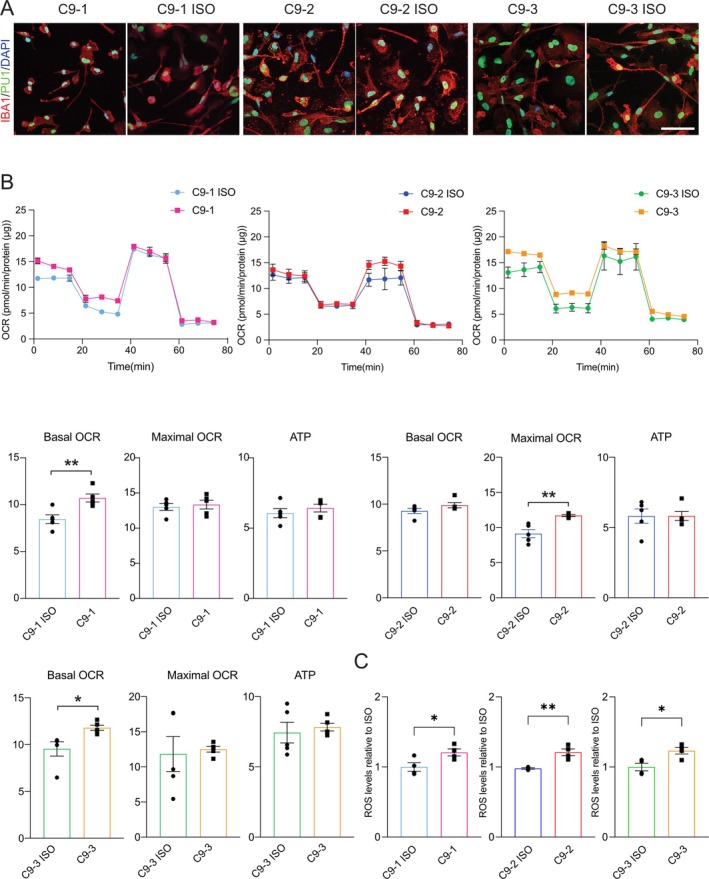
Differentiation, mitochondrial bioenergetic and reactive oxygen species characterization of *C9orf72* and paired isogenic control iPSC‐derived microglia. (A) Representative confocal images showing the immunocytochemical staining of IBA1 (red) and PU1 (green) with nuclei (DAPI, blue) in *C9orf72* (C9) and paired isogenic control (C9‐ISO) iPSC‐derived microglia. Scale bar, 100 μm. (B) Mitochondrial respiration in C9 and C9‐ISO microglia. Oxygen consumption rates (OCRs) were measured over time after the sequential addition of oligomycin, CCCP, and rotenone/antimycin A. Values for the basal OCR, ATP‐linked OCR, and maximal OCR are expressed as pmol/min/protein (μg). Mean ± SEM; unpaired two‐tailed t test; *p < 0.05 and **p < 0.01; *n* = 5 independent experiments. (C) Quantification of intracellular reactive oxygen species in C9 and C9‐ISO microglia. Mean ± SEM; unpaired two‐tailed t test; *p < 0.05 and **p < 0.01; *n* = 4 independent experiments.

To investigate the cell type‐specific impact of the *C9orf72* repeat expansion mutation on energy metabolism, we differentiated three independent *C9orf72* patient iPSC lines (BS6, C9‐1; CS29iALS, C9‐2; and CS52iALS, C9‐3) and their corresponding isogenic controls (BS6 2H9, C9‐1 ISO; CS29iALS‐Iso, C9‐2 ISO; and CS52iALS‐Iso, C9‐3 ISO) into MNs, microglia, and astrocytes (Figures 1, 2, 3). As previously reported in other studies (Hölbling et al. 2025; Lopez‐Gonzalez et al. 2016; Vahsen et al. 2023; Selvaraj et al. 2018), repeat expansion analysis in all iPSC lines confirmed variable degrees of *C9orf72* expansion (Figure S1A) alongside increased levels of the most common dipeptide repeat proteins poly‐glycine–alanine (poly‐GA) and poly‐glycine‐proline (poly‐GP) that were particularly elevated in *C9orf72* microglia compared to isogenic controls (Figure S1B). We next examined the expression of both different *C9orf72* isoforms as well as *STMN2* (full‐length and truncated) and *UNC13A* due to their association with neurodegeneration and TDP‐43 pathology. PCR analysis revealed reduced expression of different *C9orf72* isoforms alongside altered expression of truncated *STMN2* and *UNC13A* in *C9orf72* MNs compared to their corresponding isogenic controls (Figure S1C), indicating variable changes in *C9orf72* and TDP‐43 pathology‐associated transcripts across the different *C9orf72* patient lines. Furthermore, a slight decrease in *C9orf72* protein levels was observed in *C9orf72* MNs, astrocytes, and microglia (Figure S1D). Despite the *C9orf72* repeat expansion, no marked effects were observed on cell type specification (Figures 1, 2, 3, S1E).

Next, we analyzed the oxygen consumption rate (OCR) in *C9orf72* MNs, microglia, and astrocytes using Seahorse with the sequential addition of the complex V/ATP synthase inhibitor oligomycin, the protonophore CCCP, and the complex III inhibitor antimycin A to measure several parameters of mitochondrial function. We detected a significant reduction in maximal mitochondrial respiration in *C9orf72* MNs compared with isogenic controls (Figure 1B). In parallel, ATP levels were significantly lower in *C9orf72* MNs than in isogenic controls (Figures 1B, S2A). Compared with isogenic controls, *C9orf72*‐derived astrocytes and microglia presented non‐significant differences in the OCR, with a trend toward increased respiration (Figures 2B, 3B). ATP levels were significantly increased in *C9orf72*‐derived astrocytes and microglia (Figure S2A). Next, we assessed the glycolytic rate in iPSC‐derived MNs, astrocytes, and microglia. While MNs did not exhibit a significant change, *C9orf72*‐derived microglia presented a greater extracellular acidification rate (ECAR) than isogenic controls did, whereas no significant increase was observed for *C9orf72*‐derived astrocytes (Figure S2B). Given the increased glycolysis rate of *C9orf72* microglia, we further assessed intracellular reactive oxygen species using 2′,7′‐dichlorodihydrofluorescein diacetate. We identified increased oxidative stress in microglia of all three *C9orf72* iPSC lines compared to isogenic controls (Figure 3C), indicating that the *C9orf72* expansion drives a metabolic shift toward heightened oxidative stress in microglia.

### 3.2 Single‐Cell Transcriptomic and Metabolic Profiling Reveals Microglial Susceptibility to *C9orf72*‐Linked Oxidative Stress

**FIGURE 4:**
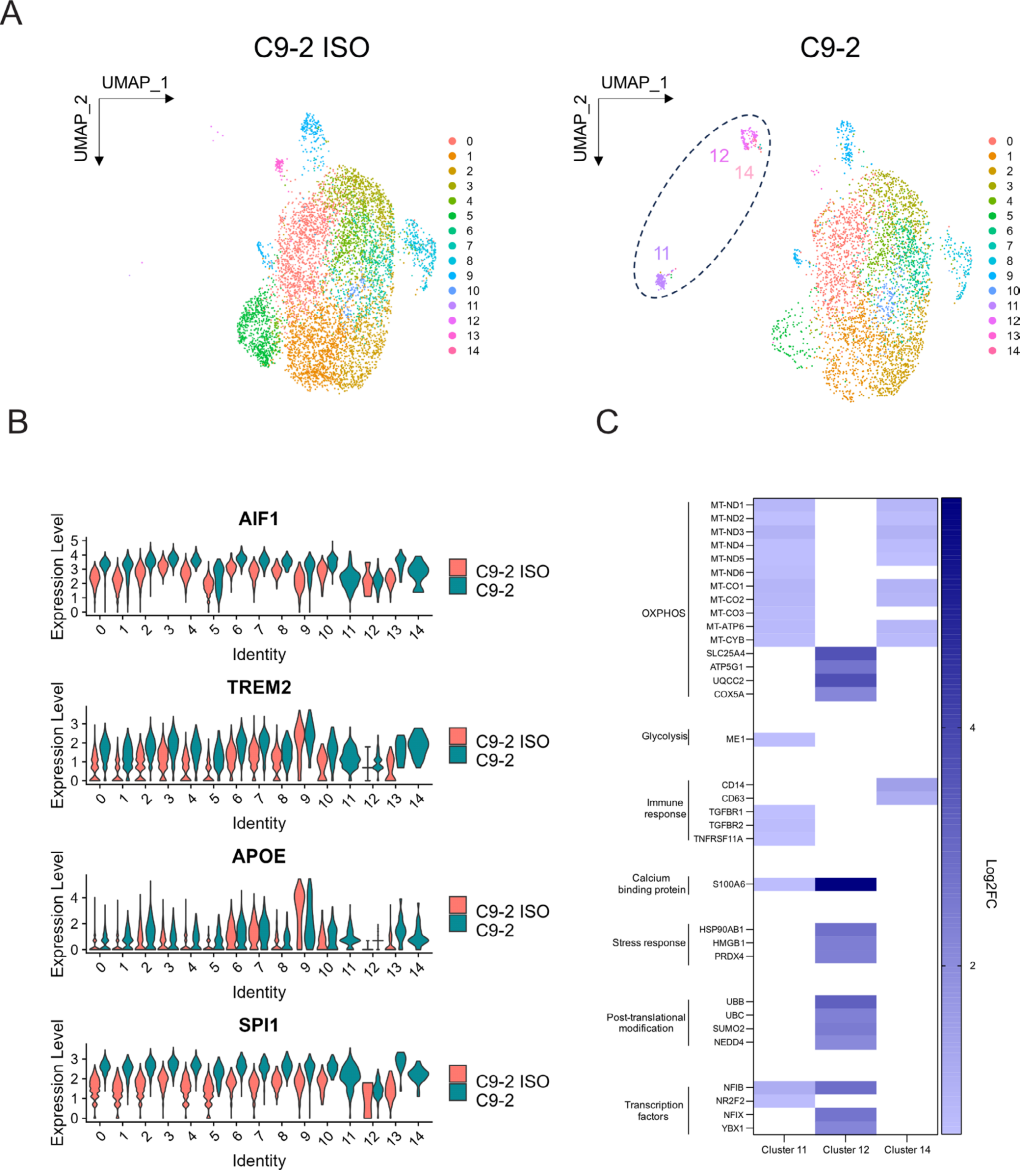
Single‐cell RNA sequencing analysis of *C9orf72* and paired isogenic control iPSC‐derived microglia. (A) UMAP plots from single‐cell RNA sequencing of C9‐2 and C9‐2 ISO iPSC‐derived microglia show comparable overall clustering, while clusters 11, 12, and 14 are uniquely present in the *C9orf72* mutant condition. (B) Violin plots display the expression of canonical microglia markers across all clusters. (C) A selection of representative genes from these clusters is shown in the heatmap organized by functional category.

To gain deeper insights into how the *C9orf72* expansion affects microglial identity and function, we performed single‐cell RNA sequencing on *C9orf72* and isogenic control iPSC‐derived microglia. While both conditions exhibited similar global clustering patterns in the UMAP (Figure 4A) and expressed canonical microglia markers (Figure 4B), microglia harboring the *C9orf72* expansion displayed distinct transcriptional clusters that were absent in control cultures, suggesting the emergence of mutant‐specific microglia subpopulations (Supporting Information, Data S1). These clusters (11, 12 and 14) were enriched for genes encoding core components of the mitochondrial respiratory chain, including genes involved in electron transport and proton pumping across the inner mitochondrial membrane (Figure 4C), supporting our findings of increased ATP levels in *C9orf72* mutant microglia. Among the most striking findings was the robust increase in *S100A6* (Figure 4C), a calcium‐binding protein linked to oxidative stress, supporting previous reports of S100A6 upregulation in other ALS models (Hoyaux et al. 2000, 2002; Leśniak et al. 2005). In addition, we observed in cluster 11 the upregulation of *ME1*, encoding malic enzyme 1, which catalyzes the oxidative decarboxylation of malate to pyruvate, contributing to NADPH production and redox balance. We further observed the marked upregulation of the antioxidant enzyme *PRDX4* in cluster 12. Additional differentially expressed genes included those involved in post‐translational modifications (*UBB*, *UBC*, *SUMO2*, *NEDD4*) and transcriptional regulation of development and stress responses (*NFIB*, *NR2F2*, *NFIX*, *YBX1*). These transcriptional alterations, alongside increased expression of inflammatory markers, such as *CD14* and *CD63* (Figure 4C), support the presence of a primed or stress‐responsive microglial state under basal conditions in *C9orf72* mutant microglia.

**FIGURE 5:**
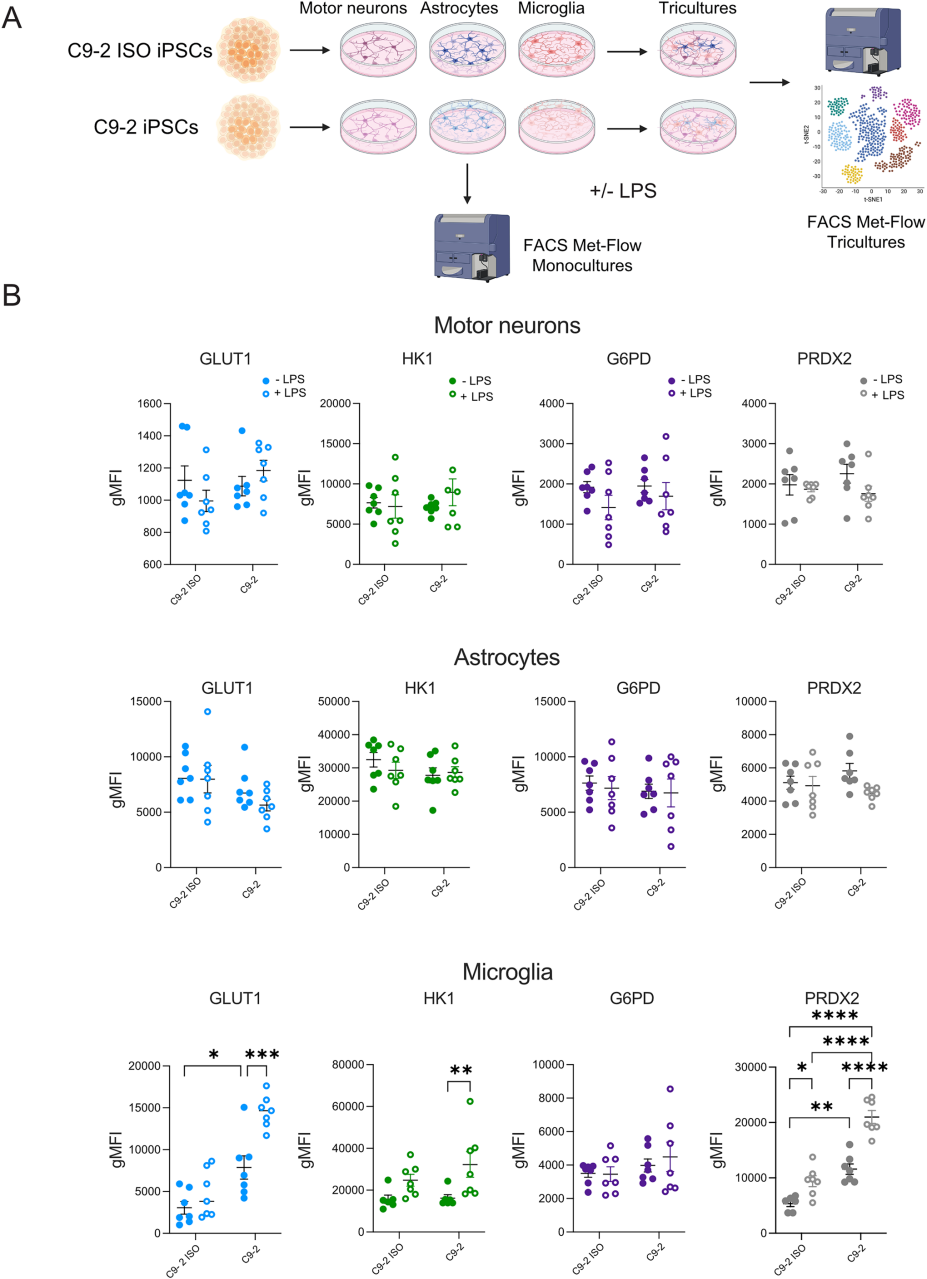
Met‐Flow‐based metabolic analysis revealed divergent metabolic profiles in *C9orf72* and paired isogenic control iPSC‐derived motor neurons, astrocytes, and microglia in monocultures. (A) Schematic of the experimental workflow. C9‐2 and C9‐2 ISO iPSCs were differentiated into motor neurons (MNs), astrocytes, and microglia. These cells were then either left untreated or treated with LPS and analyzed as monocultures or as part of a triculture system. Using Met‐Flow, we evaluated the expression of key metabolic enzymes—GLUT1, HK1, G6PD, and PRDX2—in MNs, astrocytes, and microglia derived from C9‐2 and C9‐2 ISO iPSCs. Image was created with BioRender.com. (B) Quantitative analysis of metabolic markers—GLUT1, HK1, G6PD, and PRDX2—in MNs, astrocytes, and microglia derived from C9‐2 and C9‐2 ISO iPSC lines under basal conditions and after LPS treatment. The data are expressed as the geometric mean fluorescence intensity (gMFI). Mean ± SEM; two‐way ANOVA with Bonferroni *post hoc* correction; *p < 0.05, **p < 0.01, ***p < 0.001, and ***p < 0.0001; *n* = 6 to 7 independent experiments.

To validate the metabolic features and assess the cell type‐specific impact of *C9orf72* expansion in monocultures and tricultures under basal and inflammatory conditions (Figure 5A), we used Met‐Flow, a single‐cell metabolic analysis strategy (Ahl et al. 2020). We combined a panel of cell type‐specific markers (MNs: HB9; astrocytes: CD49F, HLA‐E, GLAST; microglia: CD206, HLA‐DR, CD86, CX3CR1, CD11B, ARGI1) alongside different metabolic enzymes involved in different pathways: glucose uptake (glucose transporter 1, GLUT1), the oxidative pentose phosphate pathway (glucose 6‐phosphate dehydrogenase, G6PD), glycolysis (hexokinase‐1, HK1), and antioxidant response pathways (peroxiredoxin 2, PRDX2). Expression of cell type‐specific markers for MNs, astrocytes, and microglia remained largely unchanged in *C9orf72* mutant cells compared to their corresponding isogenic controls under basal conditions (Figure S3). However, microglia with *C9orf72* expansion showed increased expression of HLA‐DR compared to their corresponding isogenic controls (Figure S3), confirming our single‐cell RNA sequencing results of primed microglia subpopulations with *C9orf72* expansion. Regarding their metabolic profile, MNs and astrocytes from both *C9orf72* and isogenic control cultures showed no significant differences in the expression of GLUT1, HK1, G6PD, or PRDX2, suggesting that neuronal and astrocyte metabolism might be relatively unaffected by *C9orf72* expansion under basal conditions (Figure 5B). In contrast, microglia from *C9orf72* cultures presented significant metabolic changes. GLUT1 and PRDX2 expression were significantly higher in *C9orf72* microglia than in control microglia (Figures 5B, S4A), confirming increased glycolytic activity and oxidative stress. These findings suggest that microglia are particularly susceptible to metabolic disruptions associated with *C9orf72* expansion, even in the absence of external inflammatory stimuli.

### 3.3 Single‐Cell Metabolic Analysis Reveals a Role for *C9orf72* in Microglial Metabolic Reprogramming Under Inflammatory Conditions

To characterize changes in cellular metabolism under inflammatory conditions, we performed Met‐Flow analysis by quantifying rate‐limiting metabolic enzymes after LPS stimulation. Compared with the isogenic controls, in *C9orf72* microglia, LPS treatment significantly increased the expression of GLUT1, HK1, and PRDX2, indicating an enhanced metabolic and oxidative stress response (Figures 5B, S4B). *C9orf72* MNs and astrocytes did not significantly differ in GLUT1, HK1, G6PD, or PRDX2 expression after LPS stimulation (Figure 5B). These results suggest that, even though both glial and neuronal cell types express TLR4 (Lehnardt et al. 2003; Tang et al. 2007, 2008), LPS‐induced inflammation drives differential metabolic responses in MNs, astrocytes, and microglia, with the significant upregulation of the expression of key metabolic enzymes in *C9orf72* microglia. The increased expression of GLUT1, HK1, and PRDX2 in microglia suggests a potential mechanism for metabolic dysregulation and oxidative stress in response to inflammatory stimuli in neurodegenerative disease processes.

### 3.4 Neuronal‐Glial Metabolic Interactions in an iPSC‐Derived Triculture System Are Linked to Increased Cellular Metabolic Activity Relative to Monocultures

**FIGURE 6:**
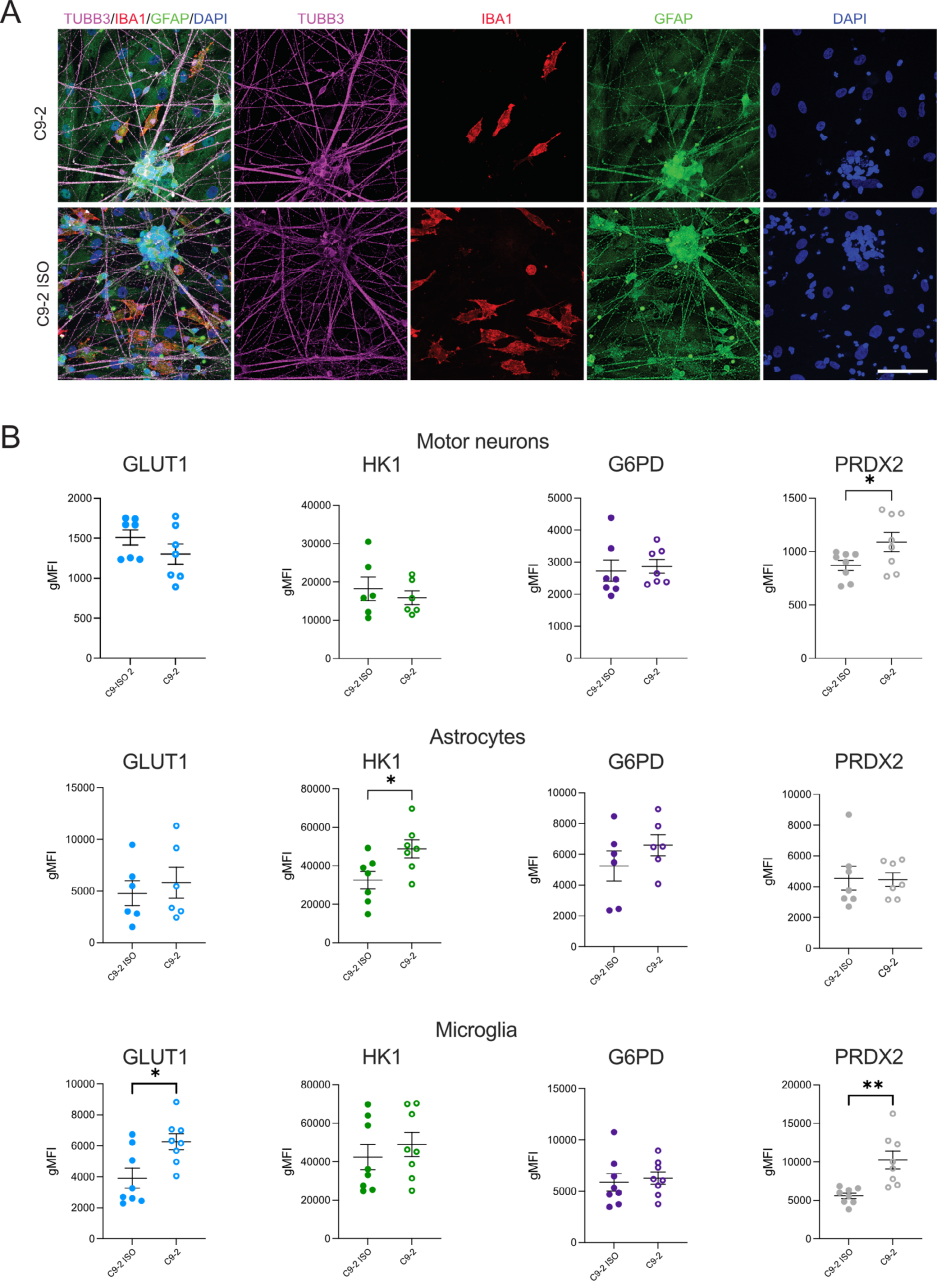
Metabolic profiling of iPSC‐derived neuronal‐glial tricultures reveals microglial susceptibility to *C9orf72*‐associated disruptions under basal conditions. (A) Representative immunofluorescence images showing the cellular composition of C9‐2 and C9‐2 ISO tricultures. The cells were stained for TUBB3 (magenta) to identify motor neurons (MNs); IBA1 (red) to identify microglia; GFAP (green) to identify astrocytes; and DAPI (blue) to identify nuclei. The individual channels for TUBB3, IBA1, and GFAP are shown to the right. Scale bar, 100 μm. (B) Met‐Flow analysis of metabolic markers in C9‐2 and C9‐2 ISO iPSC‐derived cells under basal conditions. The metabolic markers GLUT1, HK1, G6PD, and PRDX2 were assessed in MNs, astrocytes, and microglia. The data are presented as the geometric mean fluorescence intensity (gMFI) for each marker, comparing C9‐2 with C9‐2 ISO. Mean ± SEM; unpaired two‐tailed t test; *p < 0.05 and **p < 0.01; *n* = 7 independent experiments.

Neuronal‐glial interactions play a critical role in modulating inflammatory responses in the central nervous system (CNS), and changes in their interactions have been implicated in ALS (King et al. 2011). To assess the role of *C9orf72* in neuronal‐glial metabolic interactions, we developed a human iPSC‐derived triculture system. This system consists of 56% iPSC‐derived MNs, 11% astrocytes, and 33% microglia at the time of plating. Immunofluorescence imaging at day 23 confirmed the presence of MNs, astrocytes, and microglia within the triculture system (Figures 6A, S5A). Triculture differentiation medium did not affect characteristic marker expression in MNs, astrocytes, and microglia monocultures, as assessed by immunostaining for CHAT and TUBB3 for MNs, GFAP for astrocytes, and PU1 and IBA1 for microglia for both genotypes (Figure S5B–D). After 10 days of coculture, the metabolic profiles of each cell type were assessed in tricultures using Met‐Flow and cell type‐specific markers to distinguish between MNs, astrocytes, and microglia (Figure S6A,B). The cellular distribution and cell survival were comparable between *C9orf72* and isogenic control tricultures (Figure S6C,D). Compared to monocultures, MNs from isogenic control tricultures presented significantly greater expression of GLUT1, G6PD, and HK1, indicating a global increase in metabolic activity when these cells are in a multicellular environment (Figure S7). In contrast, the PRDX2 levels were lower in MNs in the triculture system than in those in the monoculture system, suggesting a reduction in oxidative stress (Figure S7). In the triculture system, astrocytes showed a significant decrease in GLUT1 expression (Figure S7). These findings suggest that astrocytes in a multicellular context may experience a slight shift in glucose metabolism but not in oxidative stress levels. Similarly, in microglia, HK1 and G6PD expression was significantly higher in tricultures than in monocultures, highlighting the increased metabolic and oxidative activity of these cells when they interact with other cell types, possibly driven by signals from neighboring MNs (Figure S7). Collectively, these findings suggest that the cellular microenvironment plays a critical role in modulating the metabolic state of iPSC‐derived MNs, astrocytes, and microglia, increasing the metabolic activity of the different cell types studied.

### 3.5 The Metabolic Profiling of iPSC‐Derived Neurons and Glia Reveals Microglial Susceptibility to *C9orf72*‐Associated Metabolic Disruptions Under Basal Conditions

To investigate the baseline metabolic profile of iPSC‐tricultures with *C9orf72* expansion in the absence of inflammatory stimuli, we utilized Met‐Flow to assess the expression levels of key metabolic markers in MNs, astrocytes, and microglia. Under basal conditions, significant metabolic differences were observed in *C9orf72*‐mutant MNs compared with their isogenic controls (Figure 6B). In MNs, the expression of PRDX2, an antioxidant enzyme, was significantly increased in *C9orf72* cultures, indicating a potential increase in oxidative stress, even in the absence of inflammatory stimuli (Figure 6B). No significant changes were detected in the expression of GLUT1, HK1, or G6PD in MNs between *C9orf72* and isogenic control conditions. Compared with those derived from isogenic controls, astrocytes derived from *C9orf72* cultures presented significant increases in HK1 levels, suggesting an alteration in glycolytic metabolism in these cells. However, no significant differences in GLUT1, G6PD, or PRDX2 levels were detected in astrocytes (Figure 6B). Microglia exhibited the most pronounced metabolic changes, with the significant upregulation of both GLUT1 and PRDX2 expression in *C9orf72* tricultures, indicating an enhanced glycolytic response and heightened oxidative stress. Those results suggest that microglia in *C9orf72*‐mutant cultures may be particularly metabolically vulnerable even in the absence of an external inflammatory trigger (Figure 6B). Overall, our findings highlight the presence of baseline metabolic alterations in iPSC‐tricultures with *C9orf72* expansion, particularly in microglia and astrocytes, which may contribute to disease pathogenesis.

### 3.6 *C9orf72* Microglia Drive Astrocytic Metabolic Changes and Contribute to Motor Neuron Loss Under Inflammatory Conditions

**FIGURE 7:**
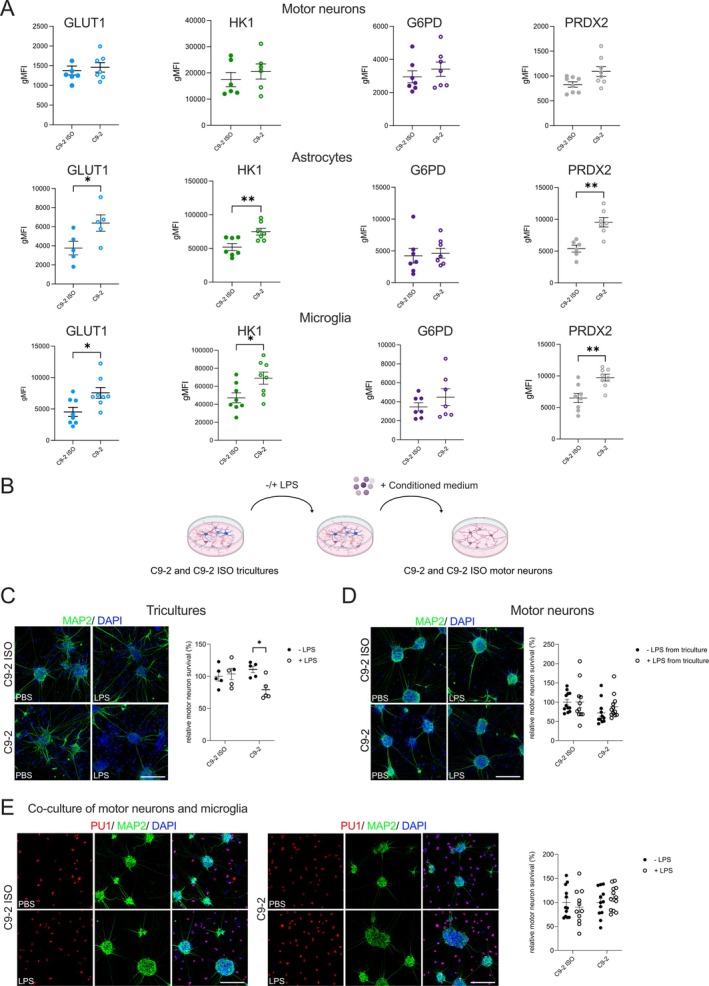
*C9orf72* microglia drive astrocytic metabolic changes and contribute to motor neuron loss under inflammatory conditions. (A) Met‐Flow analysis of metabolic markers in C9‐2 and C9‐2 ISO iPSC‐derived cells under inflammatory conditions. The metabolic markers GLUT1, HK1, G6PD, and PRDX2 were assessed in motor neurons (MNs), astrocytes, and microglia. The data are presented as the geometric mean fluorescence intensity (gMFI) for each marker, comparing C9‐2 with C9‐2 ISO upon LPS treatment. Mean ± SEM; unpaired two‐tailed t test; *p < 0.05 and **p < 0.01; *n* = 6 to 8 independent experiments. (B) Schematic of the experimental workflow. C9‐2 and C9‐2 ISO tricultures were either left untreated or treated with 100 ng/mL LPS for 24 h. MN monocultures were then exposed to conditioned medium from the corresponding LPS‐treated triculture. Image was created with BioRender.com. (C) Representative immunofluorescence images and quantification of relative MN survival in C9‐2 and C9‐2 ISO tricultures with and without 100 ng/mL LPS. Immunofluorescence images show MAP2 (green) to visualize MNs and DAPI (blue) to identify nuclei. Scale bar, 100 μm. (D) Representative immunofluorescence images and quantification of relative MN survival in C9‐2 and C9‐2 ISO monocultures with and without treatment with LPS‐treated triculture‐conditioned media. Immunofluorescence images show MAP2 (green) to visualize MNs and DAPI (blue) to identify nuclei. Scale bar, 100 μm. (E) Representative immunofluorescence images and quantification of relative MN survival in C9‐2 and C9‐2 ISO co‐cultures of MNs with microglia with and without 100 ng/mL LPS. Immunofluorescence images show PU1 (red) to identify microglia, MAP2 (green) to visualize MNs, and DAPI (blue) to identify nuclei. Scale bar, 100 μm. MN survival was calculated for each condition relative to the average value of the isogenic control under basal condition. Mean ± SEM; two‐way ANOVA with Bonferroni *post hoc* correction; *p < 0.05; *n* = 3 to 5 independent experiments.

Next, Met‐Flow analysis was conducted to examine single‐cell metabolic changes in iPSC‐tricultures containing *C9orf72* expansions compared with those in isogenic controls following LPS treatment. Quantitative metabolic analysis revealed distinct metabolic alterations in astrocytes and microglia harboring *C9orf72* expansion compared with isogenic controls (Figure 7A). In astrocytes, a significant increase in GLUT1 and HK1 was observed in *C9orf72* cells compared with isogenic controls (Figure 7A), suggesting an enhanced glycolytic response under inflammatory conditions. Furthermore, the PRDX2 levels were markedly increased, indicating a heightened oxidative stress response (Figure 7A). Similar trends were observed in microglia, where GLUT1, HK1, and PRDX2 levels were significantly elevated in *C9orf72* cells (Figure 7A), a finding that was consistent with a metabolic shift toward increased glucose metabolism and oxidative stress management. In addition to these metabolic alterations, LPS treatment led to significant changes in microglial proliferation, as evidenced by a higher percentage of KI67‐positive cells in *C9orf72* microglia compared with isogenic controls under inflammatory conditions (Figure S8). MNs did not show any detectable levels of KI67 expression. In contrast to metabolic alterations in astrocytes and microglia, MNs did not exhibit significant metabolic changes in response to LPS treatment, regardless of *C9orf72* expansion (Figure 7A). These results suggest that astrocytes and microglia, rather than MNs, may exhibit significant metabolic vulnerabilities in response to inflammatory stress in the context of *C9orf72* expansion, potentially contributing to the neurodegenerative processes observed in related pathologies. The activation of astrocytes is often correlated with the severity of disease phenotypes in mouse models of ALS, suggesting a possible microglia‐astrocyte crosstalk that regulates the astrocytic transition from neuroprotective to neurotoxic (Liddelow et al. 2017). Consistent with these findings, our data suggest that activated *C9orf72* microglia drive metabolic changes in astrocytes. To explore whether crosstalk between activated microglia and astrocytes has a neurotoxic effect on MNs, we quantified MN survival in tricultures (Figure 7B,C), MN monocultures exposed to triculture medium under basal and inflammatory conditions (Figure 7B,D), and cocultures of MNs and microglia without astrocytes (Figure 7E). Under basal conditions, MN survival did not differ between *C9orf72* and isogenic controls in the triculture system (Figure 7C). However, following LPS treatment in *C9orf72* tricultures, we observed a moderate but significant reduction in MN survival (Figure 7C). Interestingly, neither treatment of MN monocultures with conditioned medium obtained from LPS‐treated tricultures nor cocultures of MNs and microglia showed significant alterations in MN survival (Figure 7D,E), indicating that this neurotoxic effect is mediated by mechanisms dependent on cell–cell contact.

## 4 Discussion

Although microglial activation is a well‐established feature of ALS and FTD, the underlying mechanisms driving this activation remain poorly understood. This study sheds light on the metabolic vulnerabilities associated with *C9orf72* hexanucleotide repeat expansion in ALS, emphasizing the distinct, cell type‐specific effects on MNs, astrocytes, and microglia. Our findings underscore the critical role of intercellular interactions and metabolic regulation in ALS pathogenesis, offering new perspectives for the development of targeted therapeutic strategies. In this context, Met‐Flow, a single‐cell metabolic profiling tool, has proven invaluable for dissecting the intricate interplay of cell type‐specific metabolic dysregulation (Ahl et al. 2020). By enabling high‐resolution analysis of metabolic states at the single‐cell level, Met‐Flow has helped to bridge key gaps in our understanding of metabolic crosstalk between glial cells and neurons. These insights highlight promising therapeutic targets aimed at restoring cellular homeostasis, dampening neuroinflammation, and ultimately mitigating disease progression.

A key observation was the distinct, cell type‐specific metabolic impact of the *C9orf72* mutation. While MNs displayed impaired mitochondrial respiration and reduced ATP production, hallmarks of energy metabolism deficits, microglia showed a contrasting profile, characterized by increased glycolytic activity and elevated oxidative stress. These findings indicate that *C9orf72* loss of function induces differential metabolic reprogramming across brain cell types. Notably, the robust upregulation of GLUT1 and PRDX2 in microglia, both at baseline and following inflammatory stimulation, highlights their heightened sensitivity to metabolic and oxidative stress. Consistent with these functional changes, single‐cell transcriptomic analysis revealed that *C9orf72* microglia exhibit unique transcriptional signatures compared to isogenic controls, marked by elevated expression of genes involved in mitochondrial electron transport, inflammatory pathways, and overexpression of genes associated with oxidative stress (Hoyaux et al. 2000, 2002; Leśniak et al. 2005). Together, these findings point to a primed or stress‐responsive state in *C9orf72* mutant microglia, even under unstimulated conditions. This is particularly noteworthy given the high expression of *C9orf72* in myeloid cells, which play key roles in antigen presentation and immune regulation (Limone et al. 2024). Consistent with these findings, previous studies have shown that *C9orf72* deficiency induces hyperactive autoimmune responses and excessive cytokine release in bone marrow‐derived macrophages (O'Rourke et al. 2016; Burberry et al. 2016; Atanasio et al. 2016). Moreover, *C9orf72*‐mutant microglia have been reported to exhibit a proinflammatory phenotype (Vahsen et al. 2023).

Interestingly, our results suggest that the *C9orf72* mutation does not uniformly affect metabolism in all cell types but instead triggers context‐dependent metabolic responses that vary depending on both the cell type and the environmental conditions. To investigate those dynamics, we developed a novel triculture system comprising MNs, astrocytes, and microglia. This model closely mimics cellular interactions within the CNS, allowing us to dissect the intricate crosstalk among these cell types. By promoting cell–cell interactions, the triculture system provides a powerful platform for studying the specific effects of the *C9orf72* mutation on cellular metabolism in a more physiologically relevant context. Our results indicated that the triculture system not only enhanced the metabolic activity of MNs compared with monocultures but also highlighted how the mutation differentially affects the metabolic profiles of each cell type. For example, while MNs in monocultures presented significant mitochondrial deficits, they presented increased metabolic activity in the triculture system, suggesting a potential compensatory effect mediated by neuronal‐glial interactions. Similarly, astrocytes in the triculture system exhibited increased glucose metabolism, as evidenced by the upregulation of GLUT1 expression, suggesting that the multicellular environment modulates their metabolic state. Interestingly, recent data show that the partial reduction of astrocytic GLUT1 paradoxically improves glucose utilization in the brain, leading to increased neuronal activity without inducing metabolic stress (Ardanaz et al. 2024). Triculture systems may therefore enhance this beneficial astrocytic metabolic feature.

These findings support the growing concept that neuronal‐glial crosstalk plays a critical role in shaping the metabolic and functional properties of CNS cells in both health and disease (King et al. 2011). Importantly, this approach highlights the need to study neurodegenerative diseases in complex, multicellular environments to better understand the interplay between genetic and metabolic factors in disease progression. The metabolic changes observed in microglia are particularly noteworthy in the context of ALS‐associated neuroinflammation. The significant upregulation of the expression of glycolytic enzymes (e.g., GLUT1 and HK1) and oxidative stress markers (e.g., PRDX2) in *C9orf72* microglia upon LPS stimulation suggests that these cells undergo metabolic reprogramming in response to inflammatory signals. This metabolic shift likely exacerbates oxidative stress and contributes to the neurotoxic environment observed in ALS. The ability of activated *C9orf72* microglia to drive astrocytic metabolic changes underscores the potential role of microglia‐astrocyte crosstalk in disease progression. This interaction may facilitate the transition of astrocytes from a neuroprotective phenotype to a neurotoxic phenotype, as previously reported in ALS models (Liddelow et al. 2017; Nagai et al. 2007). Importantly, the increased metabolic activity in astrocytes and microglia appears to precede MN loss, suggesting that these glial cells may play a causative role in neurodegeneration.

Our triculture system revealed that the presence of *C9orf72* mutant microglia and astrocytes significantly heightened the vulnerability of MNs to inflammatory stimuli. Notably, this increased susceptibility was mediated through direct cell–cell contact and was only observed in the presence of astrocytes, underscoring their central role in driving neurotoxicity. These findings reinforce the non‐cell‐autonomous hypothesis of ALS, wherein glial cells contribute actively to MN degeneration via metabolic and inflammatory mechanisms (Ilieva et al. 2009). Moreover, our data emphasize the critical role of neuron–glia interactions in the initiation and progression of neurodegenerative processes. Specifically, we observed that activated microglia can induce the transformation of astrocytes into a reactive, proinflammatory state, thereby amplifying neurotoxic signaling cascades. This cascade is particularly relevant in the context of chronic inflammation. Consistent with our findings, previous coculture studies using iPSC‐derived microglia and MNs have shown that unstimulated *C9orf72* microglia exhibit a dysregulated cytokine and chemokine profile in their supernatant but do not directly impair MN viability or function under basal conditions. However, upon chronic LPS priming, these microglia initiate apoptotic signaling pathways in healthy MNs and drive overt neurodegeneration (Vahsen et al. 2023). Similarly, a previous study has shown that only following an excitotoxic insult, *C9orf72* microglia drive enhanced MN death (Banerjee et al. 2023). Taken together, these results suggest that *C9orf72* microglia exert neurotoxic effects primarily under conditions of sustained inflammatory stress or through complex intercellular interactions that involve a metabolic reprogramming of astrocytes. These insights further highlight the importance of glial cell dynamics in ALS pathogenesis and the potential therapeutic value of targeting microglial activation and astrocytic reactivity.

While our study provides valuable insights, it also raises several questions. First, the mechanisms by which the *C9orf72* mutation drives metabolic reprogramming in microglia and astrocytes remain incompletely understood. Given the known role of *C9orf72* in mitochondrial function (Wang et al. 2021; Choi et al. 2019), perturbations in these pathways may contribute to the observed metabolic changes. However, further studies are needed to elucidate the molecular links between *C9orf72* dysfunction and immune metabolic reprogramming. Second, our findings highlight the importance of the cellular microenvironment in modulating the effects of the *C9orf72* mutation. The triculture system used in this study provides a valuable platform for studying these interactions, but additional models that incorporate more complex features of the CNS, such as blood–brain barrier components or peripheral immune cells, may offer further insights. Finally, our study focused on basal and LPS‐induced inflammatory conditions. Future investigations should explore how other pathological stimuli, such as oxidative stress or protein aggregation, influence the metabolic and functional properties of *C9orf72* mutant cells. Such studies may help identify common pathways that could be targeted therapeutically across different ALS subtypes.

## 5 Conclusions

In conclusion, our findings reveal a critical role for *C9orf72* in modulating the metabolic and inflammatory states of MNs, astrocytes, and microglia. The cell type‐specific metabolic changes observed in this study highlight potential therapeutic targets for ALS, particularly those aimed at restoring metabolic homeostasis and mitigating oxidative stress in microglial cells. By advancing our understanding of the interplay among metabolism, inflammation, and intercellular communication in ALS, this work provides a foundation for the development of novel strategies to slow or halt disease progression.

## Author Contributions

M.D. conceived the project. M.D., M.M., I.H., C.W., C.G., and F.P. designed the experiments and interpreted the results. M.M., I.H., C.W., C.G., M.J.P., M.D., M.R., L.O., T.F.G., and U.S. performed and analyzed the data. M.M. drafted the first draft of the figures and manuscript with input from the other authors. M.M., C.W., and M.D. wrote the paper. All authors read and approved the final version of the manuscript.

## Conflicts of Interest

The authors declare no conflicts of interest.

## Supporting information


**Data S1:** Supporting Information.

**Data S2:** Supporting Information.

## Data Availability

The data that support the findings of this study are openly available in GEO at https://www.ncbi.nlm.nih.gov/geo/query/acc.cgi?acc=GSE301532, reference number GSE301532.
