# Supplementary material for: C9orf72 Repeat Expansion Induces Metabolic Dysfunction in Human iPSC‐Derived Microglia and Modulates Glial‐Neuronal Crosstalk
Source: Glia. 2025 Sep 1;74(1):e70080. doi: 10.1002/glia.70080 (PMC12667002; doi:10.1002/glia.70080)
Supplement: Supplementary file 2 — Data S2: Supporting Information. [file GLIA-74-0-s002.pdf]

Supplementary figure legends

Supplementary Figure 1. C9orf72 pathology in iPSCs, motor neurons, astrocytes, and microglia.

A) Electropherograms of the *C9orf72* hexanucleotide repeat expansion assessed by repeat-primed PCR in *C9orf72* (C9) iPSCs and their corresponding isogenic controls (C9 ISO). **B)** Quantification of dipeptide repeat proteins (in arbitrary units) in C9-2 and C9-2 ISO MNs, astrocytes, and microglia. Mean ± SEM; unpaired two-tailed t test; **P<0.01 and ***P<0.001; n=1-3 independent experiments. **C)** *C9orf72* transcript variants (3 different isoforms), stathmin-2 (*STMN2*, both full-length and truncated isoforms), and *UNC13A* were assessed by PCRs in C9-1, C9-2, and C9-3 MNs and their corresponding C9 ISO. Mean ± SEM; unpaired two-tailed t test; *P<0.05, **P<0.01, ***P<0.001, and ****P<0.0001; n=3 independent experiments. **D)** Western blot for C9orf72 expression in MNs, astrocytes and microglia for all three C9 lines and their corresponding C9 ISO. **E)** Quantification of cell type-specific marker expression in MNs (HB9), astrocytes (GFAP) and microglia (PU1) in relation to the number of nuclei (DAPI) for C9-1, C9-2, and C9-3 compared to their corresponding C9 ISO. Mean ± SEM; unpaired two-tailed t test; n=3 independent experiments.

Supplementary Figure 2. ATP concentration and extracellular acidification rate measurements in *C9orf72* and paired isogenic control motor neurons, astrocytes, and microglia.

A) ATP concentration (relative to that of the control) measured in MNs, astrocytes, and microglia. Each panel compares C9-1, C9-2, and C9-3 to their corresponding C9-ISO. The amount of ATP in each sample was calculated from standard curves and normalized to the total protein concentration. Mean ± SEM; unpaired two-tailed t test; *P<0.05 and **P<0.01; n=5 independent experiments. **B)** Extracellular acidification rate (ECAR) measurements in MNs, astrocytes, and microglia, indicating glycolytic flux over time (in minutes). Comparisons are made between C9-1, C9-2, and C9-3 and their corresponding C9 ISO. Mean ± SEM; two-way ANOVA with Bonferroni *post hoc* correction; *P<0.05; n=5 independent experiments.

Supplementary Figure 3. Characterisation of *C9orf72* and paired isogenic control motor neurons, astrocytes, and microglia monocultures under basal conditions.

Quantification of expression of cell type-specific markers in C9-2 and C9-2 ISO MNs (HB9), astrocytes (CD49F, HLA-E, GLAST), and microglia (CD206, HLA-DR, CD86, CX3CR1, CD11B, ARGI1). Mean ± SEM; unpaired two-tailed t test; *P<0.05; n=2-7 independent experiments.

Supplementary Figure 4. High-resolution Met-Flow single-cell metabolic analysis of *C9orf72* and paired isogenic control microglia.

A) Representative t-SNE plots depicting the expression levels of PRDX2 in C9-2 and C9-2 ISO microglia under basal conditions. **B)** Representative t-SNE plots depicting GLUT1 and G6PD expression in C9-2 microglia under basal conditions and after 100 ng/mL LPS stimulation. The color gradient from green to red represents the intensity of expression, with green indicating lower expression and red indicating higher expression.

Supplementary Figure 5. Characterisation of *C9orf72* and paired isogenic control iPSC-derived neuronal-glial tricultures and monocultures after treatment with triculture medium.

A) Representative immunofluorescence images showing the cellular composition of C9-2 and C9-2 ISO tricultures. The cells were stained for MAP2 (green) to identify MNs, IBA1(red) to identify microglia, and S100B (magenta) to identify astrocytes. Nuclei were identified using DAPI staining (blue). Scale bars, 100 µm. **B)** Representative immunofluorescence images showing C9-2 and C9-2 ISO MN monocultures after exposure to triculture medium. MNs were stained for TUBB3 (red) and CHAT (green). Nuclei were identified using DAPI staining (blue). Scale bar, 50 µm. **C)** Representative immunofluorescence images showing C9-2 and C9-2 ISO astrocyte monocultures after exposure to triculture medium. Astrocytes were stained for GFAP (red) and Phalloidin (green). Nuclei were identified using DAPI staining (blue). Scale bar, 50 µm. **D)** Representative immunofluorescence images showing C9-2 and C9-2 ISO microglia after exposure to triculture medium. Microglia were stained for IBA1 (red) and PU1 (green). Nuclei were identified using DAPI staining (blue). Scale bar, 50 µm.

Supplementary Figure 6. Met-Flow metabolic analysis of *C9orf72* and paired isogenic control iPSC-derived neuronal-glial tricultures.

A) Gating strategy used to perform Met-Flow metabolic analysis of iPSC-derived astrocytes, MNs, and microglia in C9-2 and C9-2 ISO tricultures. Representative scatter plots illustrate the sequential gating approach in C9-2 (bottom row) and C9-2 ISO neuronal-glial tricultures (top row). Cells were first gated for viability and singlets, followed by cell type identification: microglia (CD11B+), astrocytes (CD49F+) and MNs (CellTrace+). MNs were further distinguished by the absence of both CD11B and CD49F expression. The scatter plots show the forward scatter (FSC-A) and CellTrace parameters, allowing a clear separation of microglia, astrocytes and MNs. **B)** t-SNE plots of phenotypic markers showing the spatial distribution of astrocytes (orange), microglia (blue) and MNs (green) on the basis of CD49F, CD11B and CellTrace markers, confirming cell identity and purity. **C)** Quantification of the cellular distribution in C9-2 and C9-2 ISO tricultures under basal and inflammatory conditions.

Graph visualises average percentage of cellular distribution. Chi-square test; n=7 independent experiments. **D)** Quantification of Zombie NIR staining in MNs, astrocytes and microglia in C9-2 and C9-2 ISO tricultures under basal and inflammatory conditions. Mean ± SEM; two-way ANOVA; n=7 independent experiments.

Supplementary Figure 7. Metabolic assessment of isogenic control iPSC-derived neuronal and glial cells in monocultures versus tricultures under basal conditions using Met-Flow.

Quantitative analysis of the metabolic markers GLUT1, HK1, G6PD, and PRDX2 in MNs, astrocytes, and microglia derived from C9-2 ISO neuronal-glial monocultures and tricultures under basal conditions. The data are expressed as the geometric mean fluorescence intensity (gMFI). Mean ± SEM; unpaired two-tailed t test; *P<0.05, **P<0.01, and ***P<0.001; n=6-8 independent experiments.

Supplementary Figure 8. Characterisation of cell proliferation in *C9orf72* and paired isogenic control iPSC-derived neuronal-glial tricultures.

A) Representative immunofluorescence images showing proliferation in C9-2 and C9-2 ISO tricultures under basal and inflammatory conditions. The cells were stained for KI67 (green) to identify cell proliferation, PU1 (red) to identify microglia, and MAP2 (cyan) to identify MNs. KI67 expression was absent in MNs. Nuclei were identified using DAPI staining (blue). Scale bar, 100 µm. **B)** Quantification of cell proliferation in microglia and astrocytes in C9-2 and C9-2 ISO tricultures under basal and inflammatory condition. Mean ± SEM; two-way ANOVA with Bonferroni *post hoc* correction; **P<0.01 and ****P<0.0001; n=3 independent experiments.

Supplementary Tables

Supplementary Table 1. Primers

**Gene/transcript variant**	**Primer sequence 5'-3'**
C9ORF72 transcript variant 1_F	TCATCTATGAAATCACACAGTGTTC
C9ORF72 transcript variant 1_R	GGTATCTGCTTCATCCAGCTT
C9ORF72 transcript variant 2_F	GCGGTGGCGAGTGGATAT
C9ORF72 transcript variant 2/3_R	TGGGCAAAGAGTCGACATCA
C9ORF72 transcript variant 3_F	CAAGAGCAGGTGTGGGTTTAGGAG
STMN2 full-length isoform_F	AGCTGTCCATGCTGTCACTG
STMN2 full-length isoform_R	GGTGGCTTCAAGATCAGCTC
STMN2 truncated isoform_F	GGACTCGGCAGAAGACCTTC
STMN2 truncated isoform_R	GCAGGCTGTCTGTCTCTCTC
*UNC13A*_F	GGACGTGTGGTACAACCTGG
*UNC13A*_R	GTGTACTGGACATGGTACGGG
*GAPDH*_F	GTTCGACAGTCAGCCGCATC
*GAPDH*_R	GGAATTTGCCATGGGTGGA

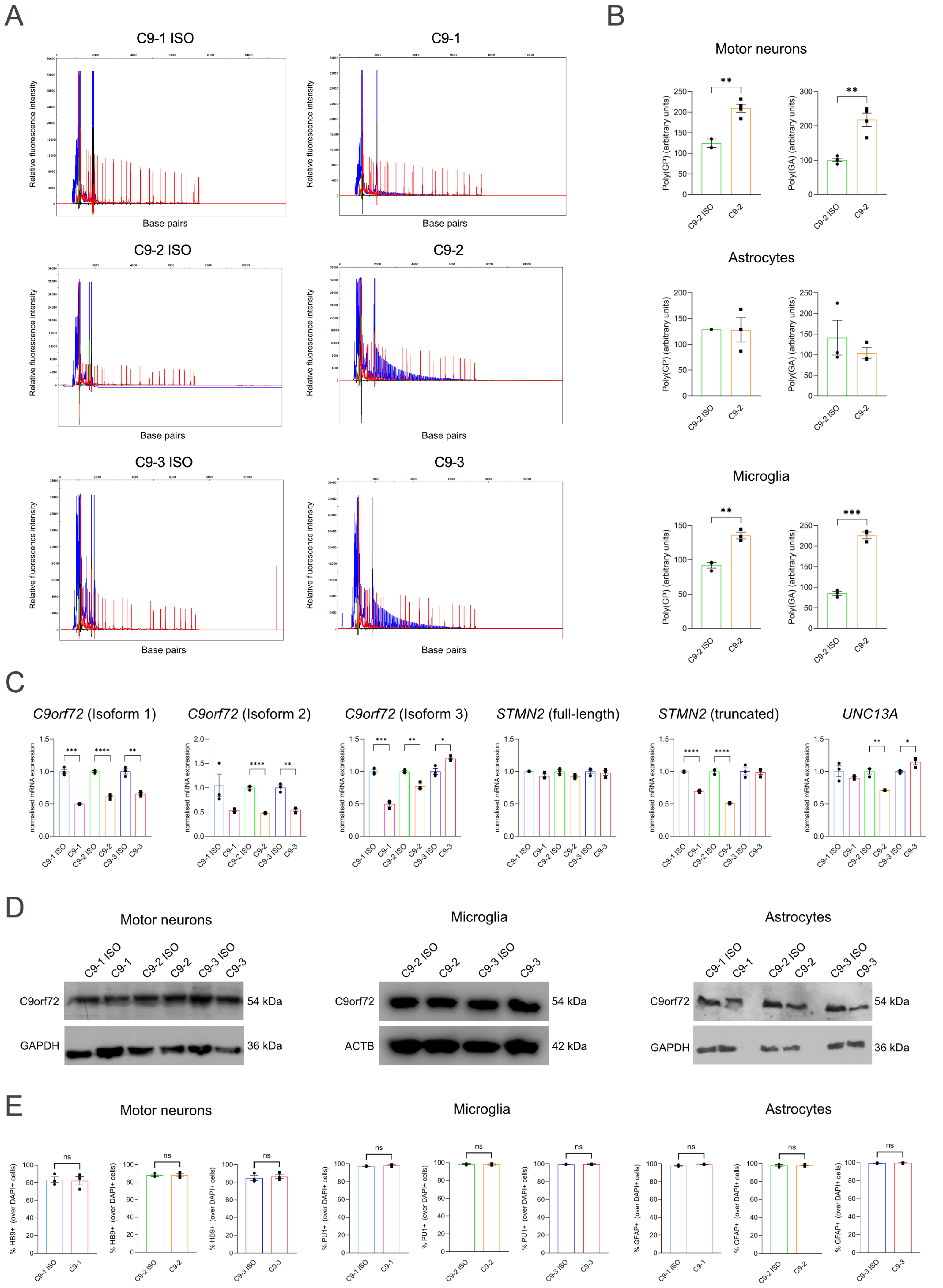

Supplementary Figure 1

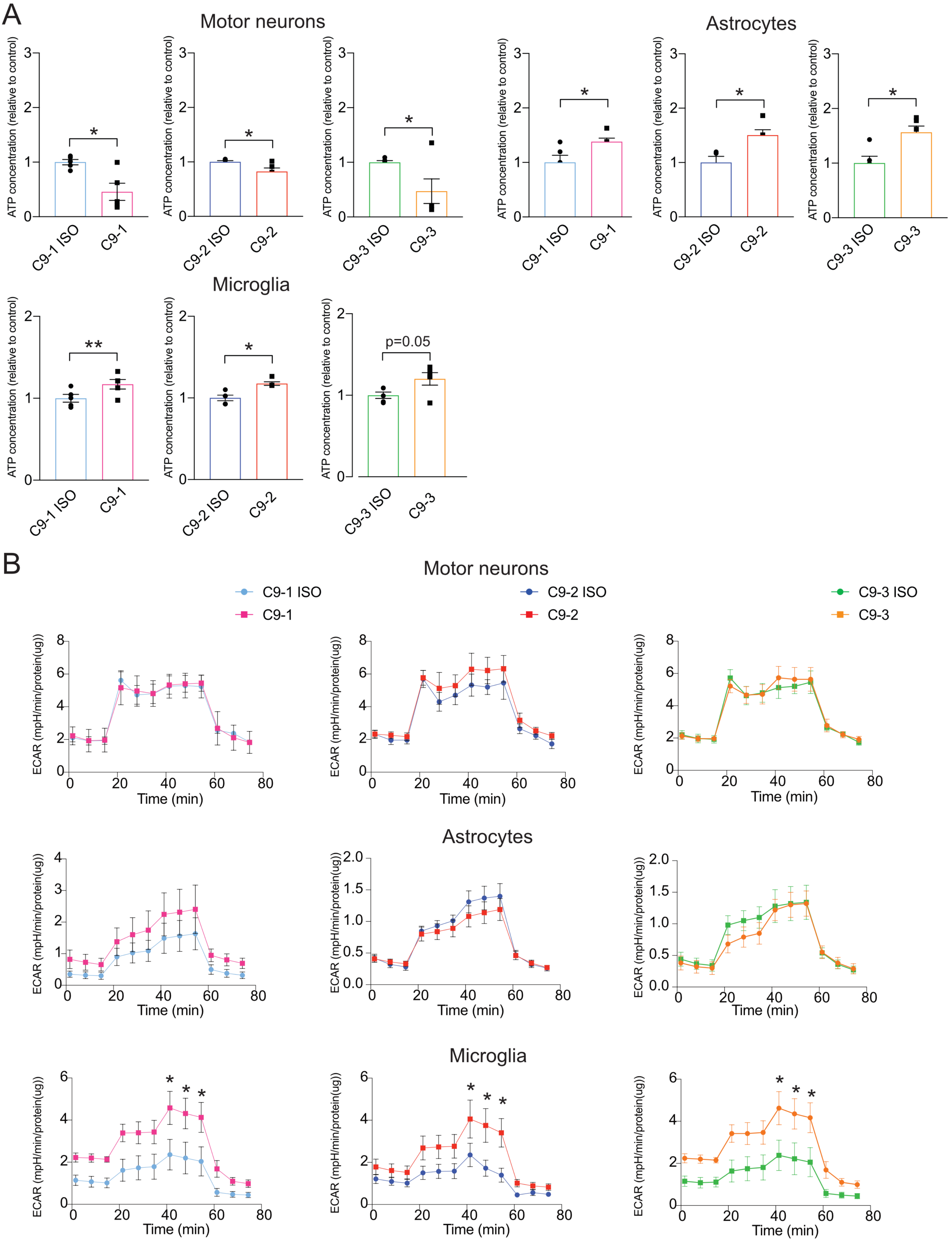

Supplementary Figure 2

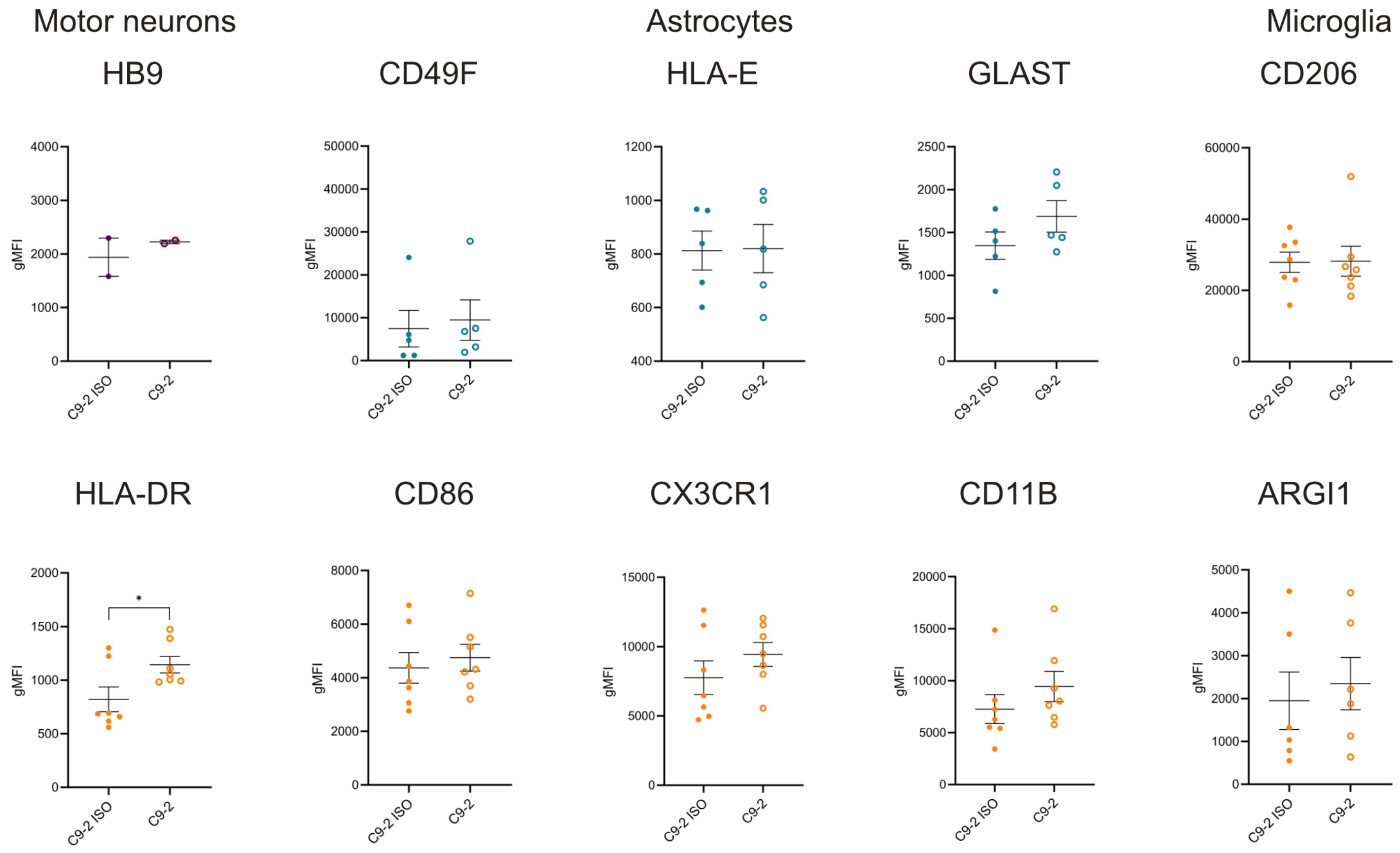

Supplementary Figure 3

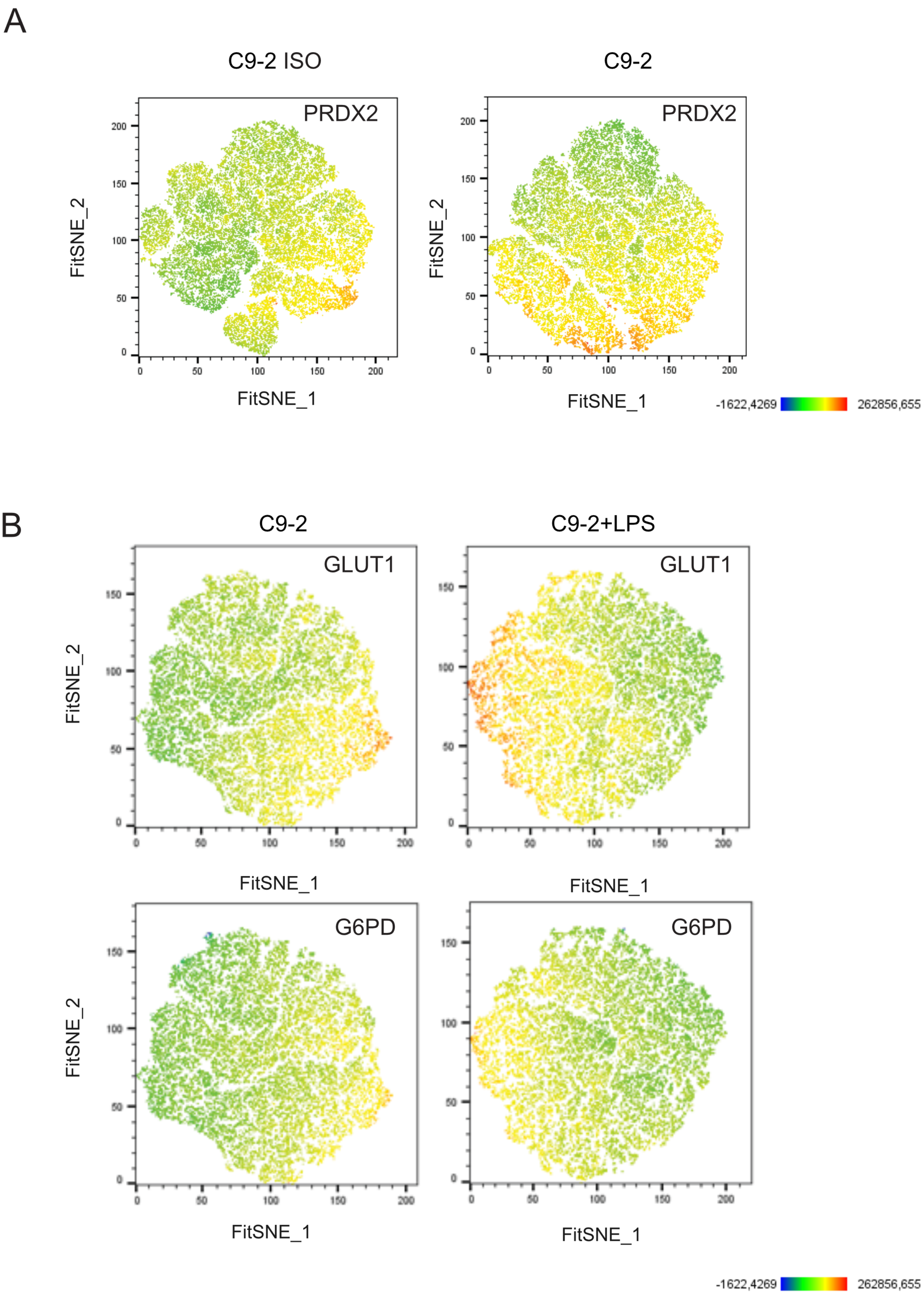

Supplementary Figure 4

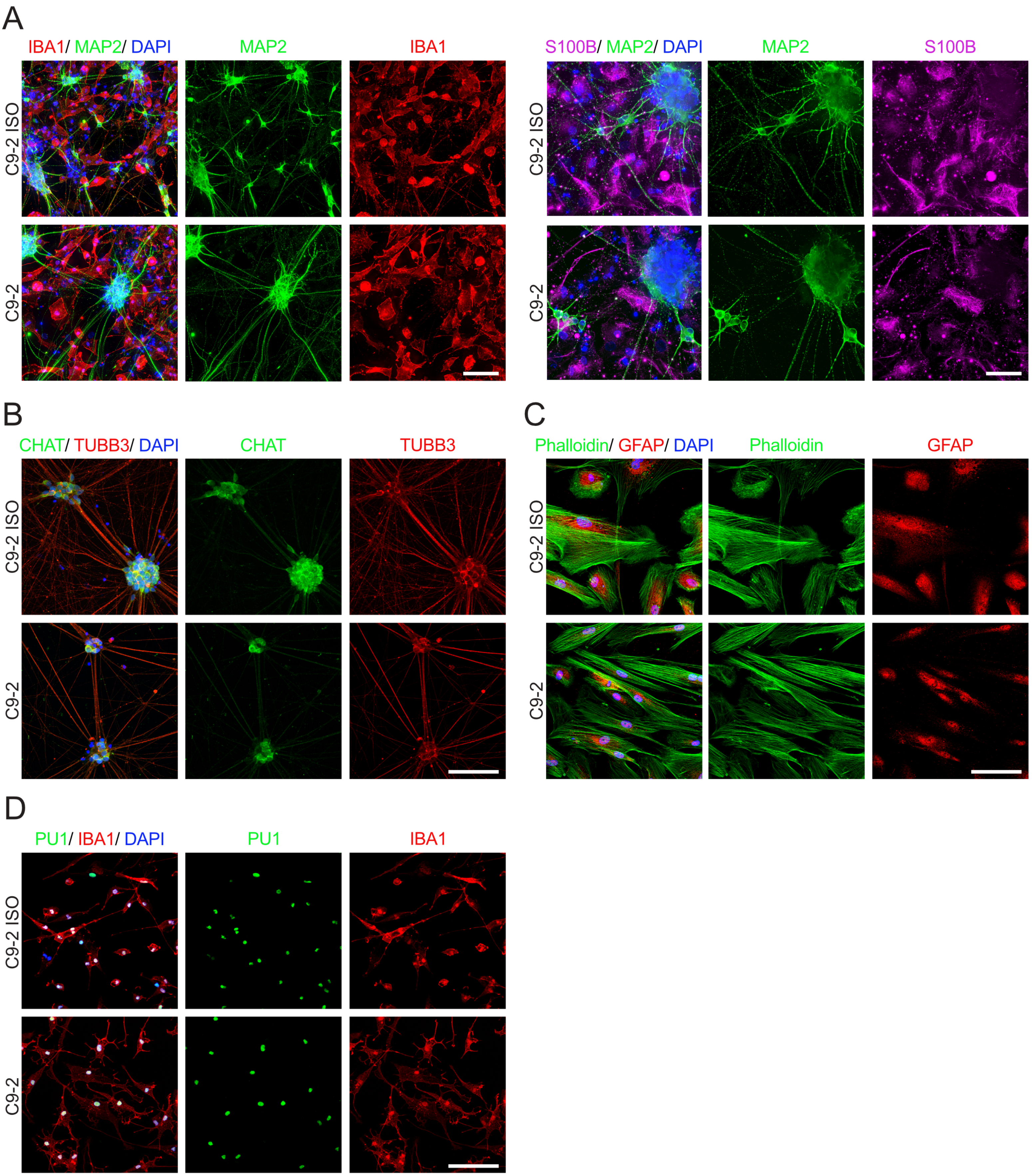

Supplementary Figure 5

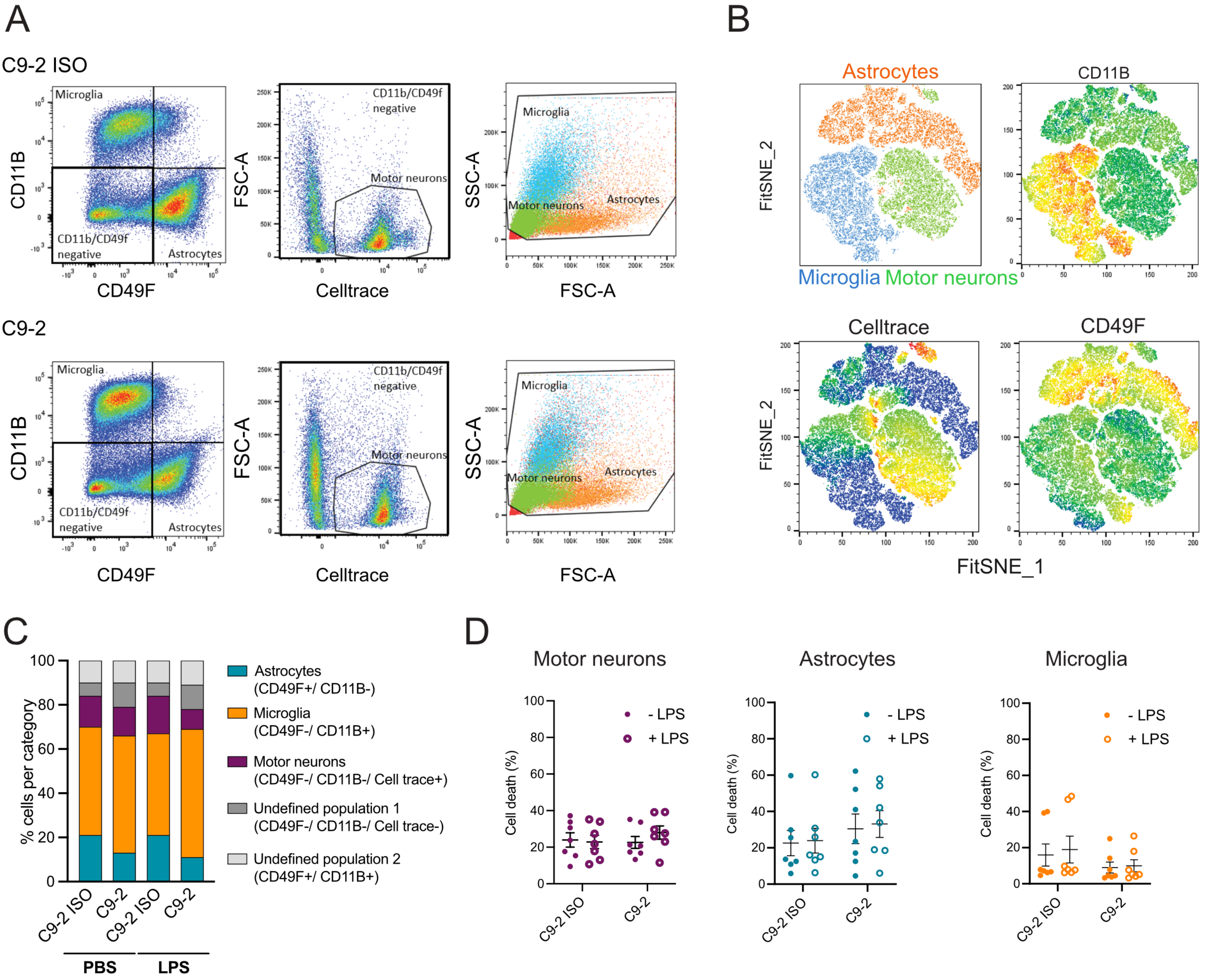

Supplementary Figure 6

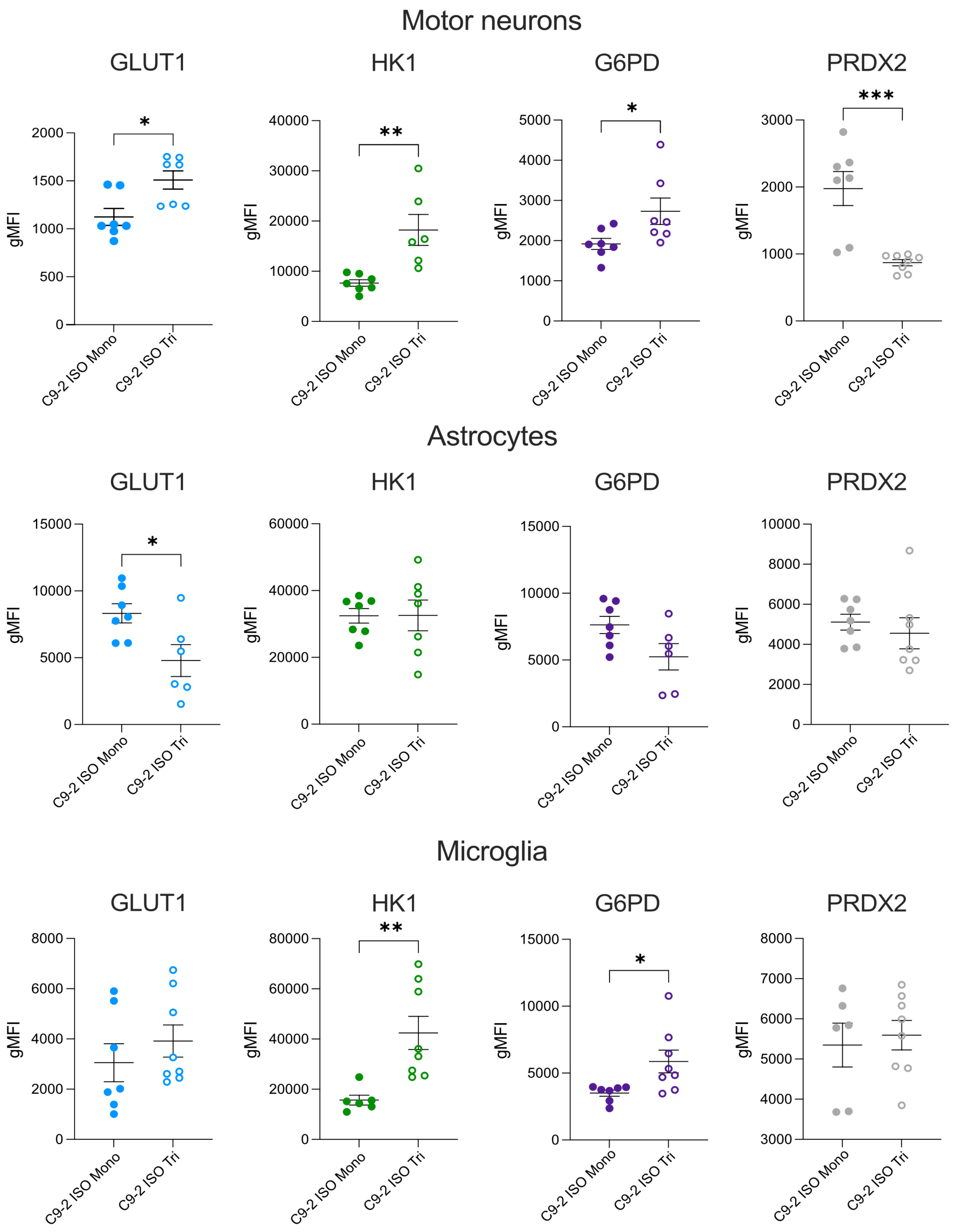

Supplementary Figure 7

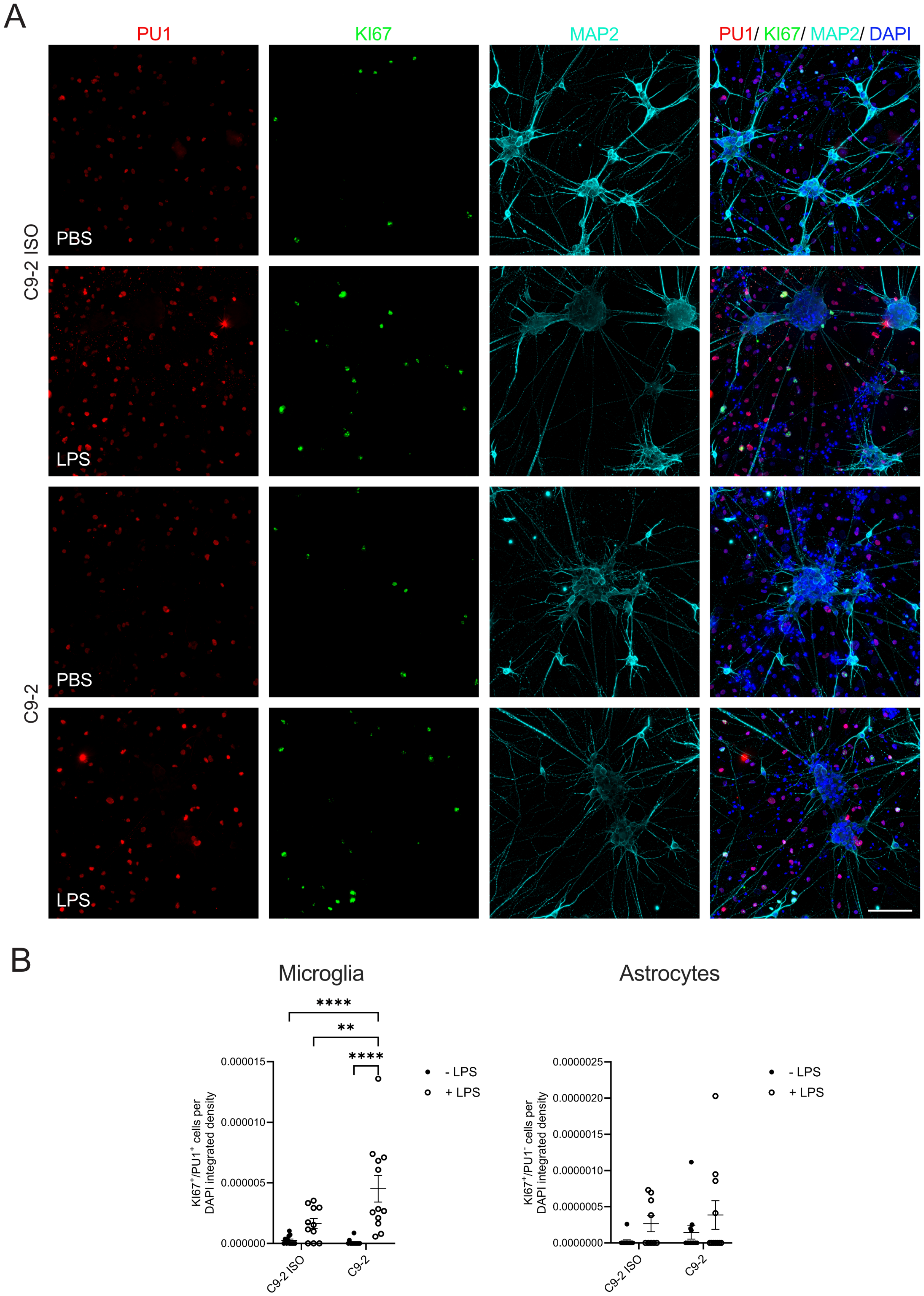

Supplementary Figure 8
